# Supplementary material for: Specific and nondisruptive interaction of guanidium-functionalized gold nanoparticles with neutral phospholipid bilayers
Source: Commun Chem. 2021 Jun 18;4:93. doi: 10.1038/s42004-021-00526-x (PMC9814519; doi:10.1038/s42004-021-00526-x)
Supplement: Supplementary file 1 — Supplementary Information [file 42004_2021_526_MOESM1_ESM.pdf]

Specific and nondisruptive interaction of guanidium-functionalized gold nanoparticles with neutral phospholipid bilayers

Lucía Morillas-Becerril,[‡,a] Sebastián Franco-Ulloa,[‡,†,b] Ilaria Fortunati,[a] Roberto Marotta,[b] Xiaohuan Sun,[c] Giordano Zanoni,[a] Marco De Vivo,[*,b] and Fabrizio Mancin[*,a]

[a] Dipartimento di Scienze Chimiche, Università di Padova, via Marzolo 1, 35131 Padova, Italy.

[b] Laboratory of Molecular Modeling and Drug Discovery, Istituto Italiano di Tecnologia, Via Morego 30, 16163 Genoa, Italy.

[c] School of Chemistry and Chemical Engineering, Yangzhou University, Yangzhou, Jiangsu, 225002, P. R. China.

† Present address: Expert Analytics. Møllergata 8, 0179, Oslo, Norway.

Email: Marco.DeVivo@iit.it, fabrizio.mancin@unipd.it

Table of contents

1. Supplementary methods 2

1.1. General 2

1.2. Dyes and synthesis of thiols 1-7 3

1.3. Synthesis and purification of gold nanoparticles 10

1.4. Characterization of nanoparticles 1-7@AuNPs 11

1.5. Preparation of neutrally charged fluorogenic liposomes 23

1.6. Preparation of negatively charged fluorogenic liposomes 23

1.7. Preparation of rigidified fluorogenic liposomes 24

1.8. Characterization of the fluorogenic liposomes 24

1.9. Liposome experiments 25

2. CryoEM liposome characterization 26

3. Additional experiments on calcein loaded liposomes 27

4. Molecular dynamics simulations 35

1. Supplementary methods

1.1 General

Chemical reagents were bought from Aldrich at highest commercial quality and used without further purification. Water was purified using a Milli-Q® and water purification system. Reactions were monitored by TLC developed on 0.25 mm Merck silica gel plates (60 F254) using UV light as visualizing agent and/or heating after spraying ninhydrin. Solvents were of analytical reagent grade, laboratory reagent grade or HPLC grade.

NMR spectra in the solution state were recorded on a AVIII 500 spectrometer (500 MHz for 1H frequency). UV-Vis absorption spectra were measured in water on a Varian Cary 50 spectrophotometer with 1 cm path length quartz cuvettes. Fluorescence spectra were measured in HEPES 10 mM or HEPES 10mM, NaCl 100 mM buffer at pH 7 on a Varian Cary Eclipse fluorescence spectrophotometer. Both the spectrophotometers were equipped with thermostatted cell holders. ESI-MS were recorded on Agilent Technologies 1100 Series system equipped with a binary pump (G1312A) and MSD SL Trap mass spectrometer (G2445D SL).

The hydrodynamic particle size (Dynamic Light Scattering, DLS) and Z-potential were measured with a Malvern Zetasizer Nano-S equipped with a HeNe laser (633nm) and a Peltier thermostatic system. Measurements were performed at 25 °C in water or HEPES 10 mM or HEPES 10mM, NaCl 100 mM buffer at pH 7. Transmission electron microscopy (TEM) was recorded on a FEI Tecnai G12 microscope operating at 100 kV. The images were registered with a OSIS Veleta 4K camera. Thermogravimetric analysis (TGA) was run on 0.4 mg nanoparticle samples using a Q5000 IR instrument from 25 to 1000 °C under a continuous air flow.

Confocal images were taken using a laser scanning confocal microscope (BX51WI-FV300, Olympus) coupled to an Argon laser (IMA-101040ALS, Melles Griot) emitting laser light at 488 nm. The laser beam was scanned on 512x512 px sample area using a 60x water immersion objective (UPLSAPO60xW-Olympus). Fluorescence emission was collected through the same objective, separated from excitation light through a 490 nm longpass dichroic mirror, and recorded by the PMT with a 510 nm longpass filter. For fluorescence lifetime experiments, the sample was excited using a frequency doubled Ti:Sapphire femtosecond laser at 440 nm, 76 MHz (VerdiV5-Mira900-F Coherent), coupled with the BX51WI-FV300 confocal microscope. The emission signal was sent to a single-photon avalanche photodiode (SPAD, MPD, Italy). Before the light enters the photodiode, it passes through a 525/50 bandpass filter. The laser sync and the output of the SPAD were fed to a time-correlated single photon counting (TCSPC) electronics (PicoHarp 300, PicoQuant, Germany) for the calculation of the emission decay curve. The fitting of decay curve

was performed with the Symphotime software (PicoQuant, Germany), using a two-components exponential model. Vitrification of samples for cryo-EM was performed in liquid ethane cooled at liquid nitrogen temperature using the FEI Vitrobot Mark IV semiautomatic autoplunger. Bright field cryo-EM was run at -176 °C in a FEI Tecnai G2 F20 transmission electron microscope, working at an acceleration voltage of 200 kV and equipped, relevant for this project, with field emission gun and automatic cryo-box. The images have been acquired in low dose modality with a GATAN Ultrascan 1000 2kx2k CCD.

1.2 Dyes and synthesis of thiols 1-7

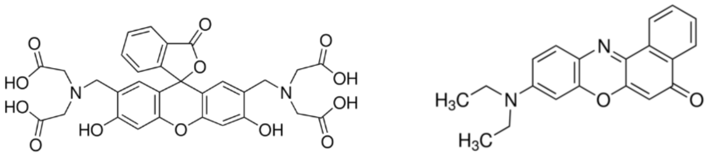

Supplementary Figure 1: Calcein (left) and nile red (right).

1.2.1.Synthesis of 2-(7-mercaptoheptyl)guanidine (thiol 1). Thiol **1** was prepared as previously reported in 5 steps:[2]

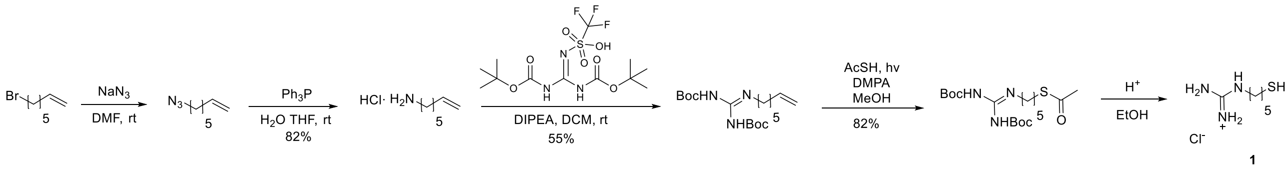

Supplementary Scheme 1: Synthesis of thiol **1**

Synthesis of 7-azidohept-1-ene. 7-bromohept-1-ene (516 mg, 3.14 mmol) and sodium azide (220 mg, 9.157 mmol) were dissolved in aqueous DMF (10 ml). After 10 hours stirring, the mixture was washed with H_2O and extracted with DCM. The combined organic layer was concentrated in vacuo and used in the next step without any purification.

Synthesis of hept-6-en-1-amine hydrochloride. 7-azido-hept-1-ene (1.179 g, 8.47 mmol) was dissolved in H_2O (5 ml) and THF (13 ml). Triphenylphosphine (4.400 g, 33.88 mmol) was then added to the mixture. The solution was stirred for 12 hours at room temperature. After the completion of the reaction, the mixture was washed with DCM and extracted with HCl solution (1 M). the combined aqueous solution was evaporated to dryness in vacuo. 785 mg (82%) were obtained as white solid. ^{1}H NMR (500 MHz, D_2O) δ 5.89 – 5.73 (m, 1H), 5.02–4.86 (dd, 2H), 2.94-2.87 (t, 2H), 2.05–1.94 (q, 2H), 1.63 – 1.50 (q, 2H), 1.40–1.24 (m, 4H). ^{13}C NMR (126 MHz, D_2O) δ 139.67, 114.32, 39.42, 32.69, 27.41, 26.47, 25.00. ESI-MS (m/z): 114.1 [$M+H^+$].

Synthesis of 1.3-bis(tert-butoxycarbonyl)-1-(hept-6-enyl)-guanidine. Hept-6-en-1-amine hydrochloride (200 mg, 1.36 mmol) and N,N'-Bis(tert-butoxycarbonyl)-N''-triflylguanidine (443.6 mg, 1.12 mmol) were dissolved in DCM. Then N,N-Diisopropylethylamine (0.592 ml, 3.4 mmol) was added to the solution. The mixture was stirred for 25 hours at room temperature. After the solvent evaporation, the crude product was purified by flash chromatography (silica gel, eluent: PE/ EtOAc 9.5:0.5). 264 mg (55%) were obtained. ^{1}H NMR (500 MHz, $CDCl_3$) δ 5.86-5.75 (m, 1H), 5.06-4.91 (m, 2H), 3.46-3.39 (q, 2H), 2.11-2.04 (q, 2H), 1.63-1.56 (m, 2H), 1.55-1.49 (d, 18H), 1.48-1.35 (m, 4H). ^{13}C NMR (126 MHz, $CDCl_3$) δ 156.09, 153.33, 138.71, 114.39, 83.04, 79.23, 40.93, 33.54, 28.83, 28.47, 28.32, 28.08, 26.30. ESI-MS (m/z): 356.2561 [M+H$^+$].

Synthesis of S-(7-((2,2,10,10-tetramethyl-4,8-dioxo-3,9-dioxa-5,7-diazaundecan-6-ylidene)amino)heptyl) ethanethioate. Carbamate derivative (110 mg, 0.309 mmol) was dissolved in methanol (3 ml). Nitrogen was injected into the solution for 30 min to remove oxygen. Afterwards, 2, 2-Dimethoxy-2-phenylacetophenone (4.0 mg, 0.015 mmol) and thioacetic acid (94.08 mg, 1.236 mmol) were added. The mixture was left under irradiation (UV, 365 nm) for 3 hours. After solvent evaporation, the crude product was purified by flash chromatography (silica gel, eluent: PE/ EtOAc 9.5:0.5). 110 mg (82%) were obtained. ^{1}H NMR (500 MHz, MeOD) δ 3.39-3.34 (t, 2H, 2.91-2.86 (t, 2H), 2.32-2.30 (s, 3H), 1.63-1.56 (m, 4H), 1.55 (s, 9H), 1.49 (s, 9H), 1.44-1.35 (m, 6H). ^{13}C NMR (126 MHz, MeOD) δ 196.15, 163.17, 156.17, 152.85, 83.04, 78.93, 40.31, 29.24, 29.09, 28.55, 28.39, 28.31, 28.22, 27.17, 26.82, 26.23.

Synthesis of 2-(7-mercaptoheptyl)guanidine. Thioacetate derivative (110 mg, 0.255 mmol) was dissolved in ethanol (5.2 ml). A 6 M HCl solution in water (5.2 ml) was added and the mixture was stirred at 78 °C for 3 hours. The reaction mixture was allowed to cool and the solvent was evaporated to obtain 70 mg (quantitative). ^{1}H NMR (500 MHz, MeOD) δ 3.23-3.17 (t, 2H), 2.54-2.48 (t, 2H), 1.65-1.56 (m, 4H), 1.48 – 1.33 (m, 6H). ^{13}C NMR (126 MHz, MeOD) δ 157.23, 41.12, 33.68, 30.05, 28.42, 28.36, 27.84, 26.2. ESI-MS (m/z): 190.1398 [M+H$^+$].

1.2.2. Synthesis of 2-(11-mercaptoundecyl)guanidine (thiol 2). Thiol **2** was prepared in 5 steps:

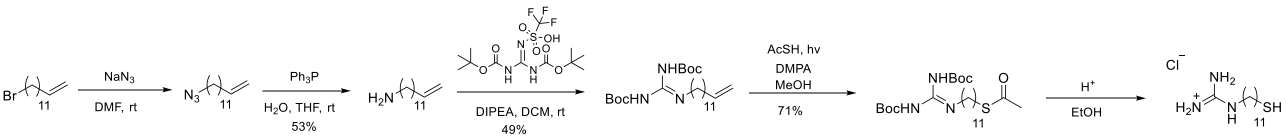

Supplementary Scheme 2: Synthesis of thiol **2**

Synthesis of 11-azidoundec-1-ene. 11-bromoundec-1-ene (1.59 g, 9.004 mmol) and sodium azide (1.17 g, 18.008 mmol) were dissolved in aqueous DMF (4 ml). After 10 hours stirring, the mixture

was washed with H_2O and extracted with DCM. The combined organic layer was concentrated in vacuo and used in the next step without any purification.

Synthesis of undec-10-en-1-amine hydrochloride. 11-azido-undec-1-ene (1.58 g, 7.58 mmol) was dissolved in H_2O (2.5 ml) and THF (10 ml). Triphenylphosphine (3.2 g, 12.13 mmol) was then added to the mixture. The solution was stirred for 12 hours at room temperature. After the solvent evaporation, the crude product was purified by flash chromatography (alumina basic, eluent: DCM/MeOH 9:1). 720 mg (53%) were obtained as white solid. ^1H NMR (500 MHz, $CDCl_3$) δ 5.87 – 5.67 (m, 1H), 4.99-4.98 (d, 1H), 4.91-4.89 (d, 1H), 2.67–2.60 (t, 2H), 2.01 – 1.94 (t, 2H), 1.40–1.24 (m, 18H). ^{13}C NMR (126 MHz, $CDCl_3$) δ 139.37, 114.28, 42.26, 33.98, 33.73, 29.64, 29.30, 29.11, 27.06.

Synthesis of 1.3-bis(tert-butoxycarbonyl)-1-(undec-10-enyl)-guanidine. Undec-10-en-1-amine hydrochloride (200 mg, 1.18 mmol) and N,N'-Bis(tert-butoxycarbonyl)-N''-triflylguanidine (383.53 mg, 0.98 mmol) were dissolved in DCM. Then N,N-Diisopropylethylamine (0.514 ml, 2.95 mmol) was added to the solution. The mixture was stirred for 25 hours at room temperature. After the solvent evaporation, the crude product was purified by flash chromatography (silica gel, eluent: PE/ EtOAc 9.5:0.5). 196 mg (49%) were obtained. ^1H NMR (500 MHz, $CDCl_3$) δ 5.86-5.75 (m, 1H), 5.06-4.91 (m, 2H), 3.46-3.39 (q, 2H), 2.11-2.04 (q, 2H), 1.63-1.56 (m, 2H), 1.55-1.49 (d, 18H), 1.48-1.35 (m, 4H). ^{13}C NMR (126 MHz, $CDCl_3$) δ 156.09, 153.33, 138.71, 114.39, 83.04, 79.23, 40.93, 33.54, 28.83, 28.47, 28.32, 28.08, 26.30. ESI-MS (m/z): 356.2561 $[M+H^+]$.

Synthesis of 1.3-bis(tert-butoxycarbonyl)-1-(5-((11-(acetylthio)undecyl)guanidine. Carbamate derivative (196 mg, 0.476 mmol) was dissolved in methanol (3 ml). Nitrogen was injected into the solution for 30 min to remove oxygen. Afterwards, 2, 2-Dimethoxy-2-phenylacetophenone (6.2 mg, 0.0238 mmol) and thioacetic acid (144.93 mg, 1.904 mmol) were added. The mixture was left under irradiation (UV, 365 nm) for 3 hours. After solvent evaporation, the crude product was purified by flash chromatography (silica gel, eluent: PE/ EtOAc 9:1). 164 mg (71%) were obtained. ^1H NMR (500 MHz, MeOD) δ 3.38-3.35 (t, 2H), 2.89-2.86 (t, 2H), 2.32 (s, 3H), 1.62-1.57 (m, 4H), 1.55 (s, 9H), 1.49 (s, 9H), 1.39-1.33 (m, 12H). ^{13}C NMR (126 MHz, MeOD) δ 196.04, 163.15, 156.14, 152.86, 83.03, 78.90, 40.42, 29.38, 29.18, 29.16, 28.89, 28.82, 28.66, 28.47, 28.41, 27.23, 26.89, 26.43.

Synthesis of 2-(11-mercaptoundecyl)guanidine. Thioacetate derivative (164 mg, 0.255 mmol) was dissolved in ethanol (7.6 ml). A 6 M HCl solution in water (7.6 ml) was added and the mixture was stirred at 78 °C for 3 hours. The reaction mixture was allowed to cool and the solvent was evaporated to obtain 104 mg (quantitative). ^1H NMR (500 MHz, MeOD) δ 3.38-3.35 (t, 2H), 2.89-

2.86 (t, 2H), 1.62-1.57 (m, 4H), 1.55 (s, 9H), 1.49 (s, 9H), 1.39-1.33 (m, 12H). ESI-MS (m/z): 246.1948 [M+H$^+$].

1.2.3. Synthesis of 1-(5-((11-mercaptoundecyl)oxy)pentyl)guanidinium chloride (thiol 3). Thiol **3** was prepared in 5 steps:

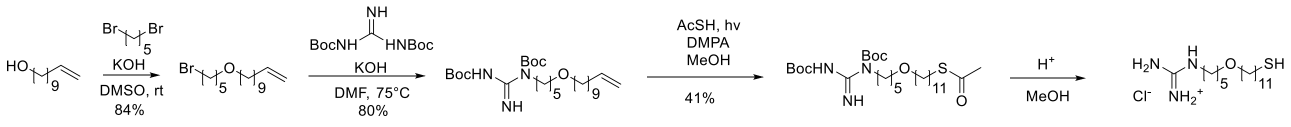

2

Supplementary Scheme 3: Synthesis of thiol **3**

Synthesis of 11-((5-bromopentyl)oxy)undec-1-ene. 11-undecen-1-ol (1.18 mL, 5.87 mmol) was dissolved in DMSO (12 mL) and KOH (1.32 g, 23.5 mmol) was then added under stirring at room temperature. After 10 minutes 1,5-dibromopentane (3.18 mL, 23.5 mmol). After 3 hours of stirring the mixture was extracted with diethyl ether (3 x 30 mL), the combined ether phases were then washed with water (3 x 30mL). After evaporation the product was purified by flash column chromatography (silica gel, eluent: gradient from petroleum ether to pet.et./EtOAC 9:1). 1.59 g (84%) of product were obtained. ^{1}H NMR (500 MHz, $CDCl_3$) δ 5.82 (ddt, 1H), 4.99 (m, 2H), 3.42 (m, 6H), 2.05 (dd, 2H), 1.90 (qn, 2H), 1.58 (m, 6H), 1.35 (m, 12H). ^{13}C NMR (126 MHz, $CDCl_3$) δ 139.22, 114.12, 71.05, 70.48, 33.83, 33.76, 32.65, 29.76, 29.55, 29.49, 29.45, 29.14, 28.93, 26.19, 24.96.

Synthesis of 1,3-bis(tert-butoxycarbonyl)-1-(5-(undec-10-en-1-iloxy)pentyl)guanidine. Guanidine hydrochloride (0.99 g, 10.4 mmol, 1eq.) and NaOH (1.66 g, 41.6 mmol) were added to a mixture of dioxane (20 mL) and water (10 mL). The solution was cooled to 0°C and Boc_2O (5.00 g, 22.9 mmol) was added. The mixture was stirred at room temperature for 36h. After evaporation, the product was purified by flash column chromatography (silica gel, eluent: DCM/MeOH 97:3) obtaining 1.471 g (57 %) of product and used for the next step. 11-((5-bromopentyl)oxy)undec-1-ene (476 mg, 1.49 mmol), 1,3-bis(terz-butoxycarbonyl)guanidine (773 mg, 2.97 mmol) and KOH (334 mg, 5.96 mmol) were dissolved in DMF (4 mL). The mixture was stirred for 24 hours at 75°C. After evaporation of the solvent the product was purified by flash column chromatography (silica gel, eluent: gradient from pet.et./EtOAc 95:5 to 23:2). 591 mg (80%) of product were obtained. ^{1}H NMR (500 MHz, $CDCl_3$) δ 9.40 (br, 1H), 5.82 (ddt, 1H), 4.96 (ddd, 2H), 3.90 (t, 2H), 3.40 (2t, 4H), 2.04 (dt, 2H), 1.59 (m, 6H), 1.53 (s, 9H), 1.50 (s, 9H), 1.42-1.23 (m, 14H). ^{13}C NMR (126 MHz, $CDCl_3$) δ 163.95, 160.70, 155.16, 139.20, 114.10, 83.46, 78.62, 71.03, 70.66, 44.60, 33.79, 29.77, 29.52, 29.48, 29.42, 29.11, 28.91, 28.57, 28.32, 27.80, 26.18, 23.38. ESI-MS (m/z): 498 [M + H$^+$].

Synthesis of 1.3-bis(tert-butoxycarbonyl)-1-(5-((11-(acetylthio)undecyl)oxy)pentyl)guanidine. 1,3-bis(tert-butoxycarbonyl)-1-(5-(undec-10-en-1-iloxy)pentyl)guanidine (150 mg, 0.3 mmol), thioacetic acid (0.23 mL, 2.8 mmol) and 2.2-dimethoxy-2-phenylacetophenone (7.8 mg, 0.03 mmol) were added to degassed methanol (3 mL) in a quartz cuvette. The mixture was irradiated with a filtered (long pass filter, 350 nm cut off) high pressure mercury lamp (100 watt) under stirring for 8 h. After evaporation the product was purified by flash column chromatography (silica gel, eluent: pet.et./EtOAc 8:2). 70 mg (41 %) of product were obtained. ^{1}H NMR (500 MHz, $CDCl_3$) δ 9.39 (br, 1H), 9.20 (br, 1H), 3.89 (t, 2H), 3.39 (t, 4H), 2.86 (t, 2H), 2.32 (s, 3H), 1.59 (m, 8H), 1.52 (s, 9H), 1.49 (s, 9H), 1.33 (m, 16H). ^{13}C NMR (126 MHz, $CDCl_3$) δ 196.08, 163.95, 160.70, 155.15, 83.46, 78.63, 71.04, 70.66, 44.58, 30.65, 29.78, 29.55, 29.50, 29.45, 29.42, 29.13, 29.10, 28.81, 28.56, 28.31, 28.03, 26.19, 23.38.

Synthesis of 1-(5-((11-mercaptoundecyl)oxy)pentyl)guanidinium chloride. 1.3-bis(tert-butoxycarbonyl)-1-(5-((11-(acetylthio)undecyl)oxy)pentyl)guanidine (70 mg) was dissolved methanol (2 mL) and hydrochloric acid (37 %, 0.2 mL) was added under stirring and nitrogen atmosphere. The solution was stirred at 77°C for 4 h. After solvent evaporation, the product was used as is for the ligand exchange.

1.2.4. Synthesis of thiol 4. Thiol 4 was prepared as previously reported in 3 steps.[1]

1.2.5. Synthesis of thiol 5. Thiol 5 was prepared as previously reported in 8 steps.[2]

1.2.6. Synthesis of 5-((11-mercaptoundecyl)oxy)-N,N,N-trimethylpentan-1-aminium bromide (thiol 6). Thiol **6** was prepared in 3 steps:

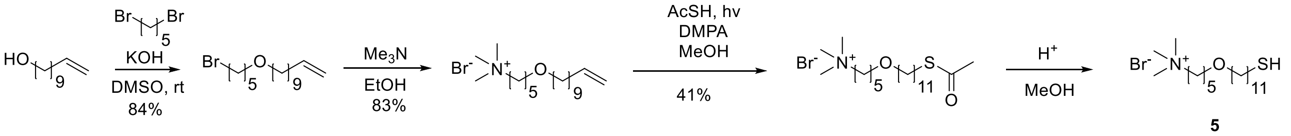

Supplementary Scheme 4: Synthesis of thiols **6**

Synthesis of 5-(undec-10-en-1-iloxy)-N,N,N-trimethylpentan-1-aminium bromide. 11-((5-bromopentyl)oxy)undec-1-ene. 11-undecen-1-ol (250 mg, 0.78 mmol) and trimethylamine (4.2 M in EtOH, 4.75 mL, 15.7 mmol) were mixed in a pressure tube and stirred at 78°C for 4 days. After solvent evaporation the product was purified by flash chromatography column (basic alumina, eluent: gradient from DCM/MeOH 24:1 to 100 % MeOH). 247 mg (83 %) of product was obtained. ^{1}H NMR (500 MHz, MeOD) δ 5.82 (ddt, 1H), 5.00 (ddd, 1H), 4.96 – 4.90 (m, 1H), 3.49 (t, 2H), 3.45 (t, 2H), 3.43 – 3.37 (m, 2H), 3.18 (s, 9H), 2.05 (m, 2H), 1.85 (m, 2H), 1.68 (m, 2H), 1.57 (m,

2H), 1.47 (m, 2H), 1.43 – 1.26 (m, 12H). ^{13}C NMR (126 MHz, MeOD) δ 138.78, 113.50, 70.68, 70.08, 66.43, 52.40, 33.50, 29.34, 29.28, 29.18, 29.15, 28.80, 28.73, 28.70, 25.86, 22.74, 22.41. ESI-MS (m/z): 298 $[M – Br]^+$.

Synthesis of 5-((11-(acetylthio)undecyl)oxy)-N,N,N-trimethylpentan-1-aminium bromide. 5-(undec-10-en-1-iloxy)-N,N,N-trimethylpentan-1-aminium bromide (225 mg, 0.59 mmol), thioacetic acid (400 µL, 5.47 mmol), 2,2-dimethoxy-2-phenylacetophenone (15.4 mg, 0.059 mmol) were dissolved in degassed MeOH (1,8 mL) in a quartz cuvette. The mixture was irradiated with a filtered (long pass filter, 350 nm cut off) high pressure mercury lamp (100 watt) under stirring for 5 h. After solvent evaporation the product was purified by flash column chromatography (basic alumina, eluent: gradient from DCM/MeOH 24:1 to 8:2) 130 mg (48 %) of product were obtained. ^{1}H NMR (500 MHz, MeOD) δ 3.48 (t, 2H), 3.45 (t, 2H), 3.41 (m, 2H), 3.19 (s, 9H), 2.88 (t, 2H), 2.33 (s, 3H), 1.85 (m, 2H) 1.68 (m, 2H), 1.57 (m, 4H), 1.48 (dt, 2H), 1.43 – 1.25 (m, 14H). ^{13}C NMR (126 MHz, MeOD) δ 196.33, 70.65, 70.02, 66.39, 52.29, 29.41, 29.36, 29.31, 29.25, 29.20, 28.82, 28.80, 28.51, 28.41, 25.91, 22.80, 22.40. ESI-MS (m/z): 374 $[M – Br]^+$

Synthesis of 5-((11-mercaptoundecyl)oxy)-N,N,N-trimethylpentan-1-aminium bromide. 5-((11-(acetylthio)undecyl)oxy)-N,N,N-trimethylpentan-1-aminium bromide (55.9 mg, 0.123 mmol) was dissolved in degassed MeOH (2 mL) and hydrochloric acid (37 %, 200 µL) was added under nitrogen atmosphere. The solution was refluxed for 6 h. The product, after evaporation, was used without further purification for the ligand exchange.

1.2.7. Synthesis of N-17-(mercapto)-N,N,N-trimethylheptadecan-1-aminium bromide (thiol 7).

Thiol **7** was prepared in 4 steps:

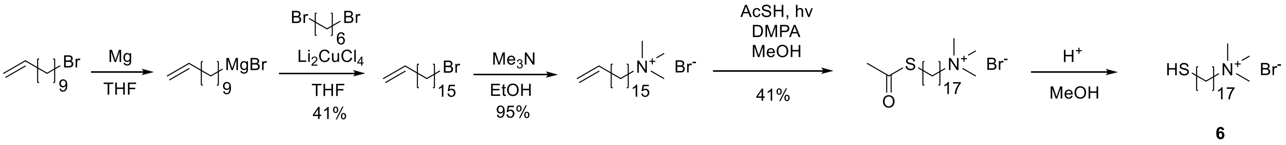

Supplementary Scheme 5: Synthesis of thiols **7**

Synthesis of 17-bromoheptadec-1-ene. Magnesium turnings (100 mg, 4.11 mmol), and dry THF (1 mL) were added to a previously dried flask. 1,2-dibromoethane (15 µL) was added to activate the magnesium. Previously vacuum-distilled 11-bromoundecene (500 µL, 3.14 mmol) was dissolved in dry THF (2 mL) and the solution was added dropwise under stirring at 50°C. After 4 hours the mixture was cooled in ice bath. A solution of $LiCuCl_4$ was prepared dissolving LiCl (5.3 mg, 0.12 mmol) and anhydrous $CuCl_2$ (8.3 mg, 0.062 mmol) in dry THF (0.6 mL), to this solution was then added 1,6-dibromohexane (967 µL, 6.29 mmol) dissolved in dry THF (2 mL). The Grignard

solution was then added dropwise to this one under stirring and nitrogen atmosphere in ice bath. After 2.5 hours the reaction was quenched with a saturated solution of NH_4Cl in water. The aqueous phase was extracted with DCM (3 x 5 mL) and the organic phases were collected and combined with the initial THF one. After solvent evaporation the product was purified by flash column chromatography (silica gel, eluent: n-hexane). 405 mg (41 %) of product were collected. ^{1}H NMR (500 MHz, $CDCl_3$) δ 5.84 (ddt, 1H), 5.01 (ddd, 1H), 4.95 (ddt, 1H), 3.43 (m, 2H), 2.06 (m, 2H), 1.87 (m, 2H), 1.51 - 1.21 (m, 24H). ^{13}C NMR (126 MHz, $CDCl_3$) δ 139.27, 114,09, 34.04, 33.84, 32.86, 29.67, 29.63, 29.56, 29.53, 29.46, 29.17, 28.96, 28.79, 28.20.

Synthesis of N,N,N-trimethylheptadec-16-en-1-aminium bromide. 17-bromoheptadec-1-ene (260 mg, 0.82 mmol) was mixed with trimethylamine solution (4.2 M in EtOH, 4.0 mL) in a pressure tube. The mixture was stirred at 78°C for 3 days. The mixture was then evaporated, and the product was purified by flash column chromatography (silica gel, eluent: DCM/MeOH from 9:1 to 8:2 + 10 mL/L Et_3N). 292 mg (95 %) of product were collected. ^{1}H NMR (500 MHz, MeOD) δ 5.82 (ddt, 1H), 5.00 (ddd, 1H), 4.93 (ddd, 1H), 3.40 (m, 2H), 3.18 (s, 9H), 2.06 (m, 2H), 1.82 (m, 2H), 1.51 – 1.23 (m, 24H). ^{13}C NMR (126 MHz, MeOD) δ 138.75, 113.44, 66.50, 52.30, 33.53, 29.40, 29.37, 29.28, 29.23, 29.21, 28.88, 28.85, 28.74, 25.99, 22.61

Synthesis of 17-(acetylthio)-N,N,N-trimethylheptadecan-1-aminium bromide. N,N,N-trimethylheptadec-16-en-1-aminium bromide (94 mg, 0.25 mmol) thioacetic acid (168 µL, 2.30 mmol) were dissolved in degassed MeOH (1.5 mL) in a quartz cuvette. The mixture was irradiated with a filtered (long pass filter, 350 nm cut off) high pressure mercury lamp (100 watt) under stirring. After 5.5 hours the mixture was evaporated and the product was purified by flash column chromatography (silica gel, eluent: gradient from DCM/MeOH 95:5 to 100 % MeOH). 66 mg (58 %) of product were obtained. ^{1}H NMR (500 MHz, MeOD) δ 3.38 (m, 2H), 3.17 (s, 9H), 2.88 (t, 2H), 2.32 (s, 3H), 1.82 (m, 2H), 1.57 (m, 2H), 1.48 – 1.25 (m, 26H). ^{13}C NMR (126 MHz, MeOD) δ 196.17, 66.48, 52.17, 29.38, 29.36, 29.29, 29.27, 29.21, 29.18, 28.86, 28.83, 28.48, 28.42, 25.98, 22.57. ESI-MS (m/z): 372.4 $[M – Br]^+$.

S***ynthesis of N-17-(mercapto)-N,N,N-trimethylheptadecan-1-aminium bromide***. Under nitrogen atmosphere 17-(acetylthio)-N,N,N-trimethylheptadecan-1-aminium bromide (55.7 mg, 0.123 mmol) was dissolved in MeOH (2 mL) and HCl (37 %, 200 µL) was added. The solution was refluxed under nitrogen atmosphere for 6 hours. After evaporation EtOH (2 mL) was added and evaporated again. The product was used as is for the nanoparticle synthesis without further purification.

1.3. Synthesis and purification of gold nanoparticles

Tetraoctylammonium bromide (TOABr, 2.5 eq) was dissolved in toluene and the solution was degassed for 40 minutes. This solution was used to wash three times an aqueous solution of gold (III) chloride trihydrate ($HAuCl_4 \cdot 3H2O$, 1 eq). The combined organic phases were collected in a round-bottom flask along with the remaining solution of TOABr. This mixture was left to stir for about 20 minutes under an inert atmosphere. Afterwards dioctylamine (DOA, 20 eq) was added all at once. After 1.5 hours the solution was put in an ice bath, then sodium borohydride ($NaBH_4$, dissolved in milli-Q water, 0.048 mg/µl, 10 eq) was added all at once. After 2 hours the drop of water (which had been used to dissolve sodium borohydride) was removed from the reaction mixture and the desired thiol dissolved in methanol was added. After the formation of the nanoparticles was observed, the mixture was usually stirred for another hour. They were purified by triturations with various organic solvents (each trituration entails the suspension of the nanoparticles in the solvent of choice, sonication, centrifugation, and then removal of the supernatant), then they were dried and characterized with various techniques.

The average formula for AuNP was calculated using the spherical approximation from the TEM average diameters of the gold cores (using the gold atom density of 59 atoms/nm^3) and the organic content obtained by TGA analyses, using the following equations:

$$n\ (gold\ atoms) = V \cdot 59\ atoms/nm^3$$

$$n\ (ligands) = \frac{\left(\frac{(\%\ weight\ loss)}{MWl_{ligand}}\right)}{\left(\frac{(\%\ weight\ residue)}{n\ (gold\ atoms) \cdot 196{,}96 g/mol}\right)}$$

$$footprint = \frac{A}{n\ (ligands)}$$

were A and V are the average surface area and volume of nanoparticle’s gold core. Thiolate footprints (the average gold surface occupied by a single thiolate) obtained were in the 0.1-0.2 nm^2 interval, which well compare with typical values of alkylthiol protected gold nanoparticles.

1.4. Characterization of nanoparticles 1-7@AuNPs

Characterization of **1'@ AuNP, 4@AuNP** and **5@AuNP** is reported elesewere.[2]

1.4.1. 1@AuNP

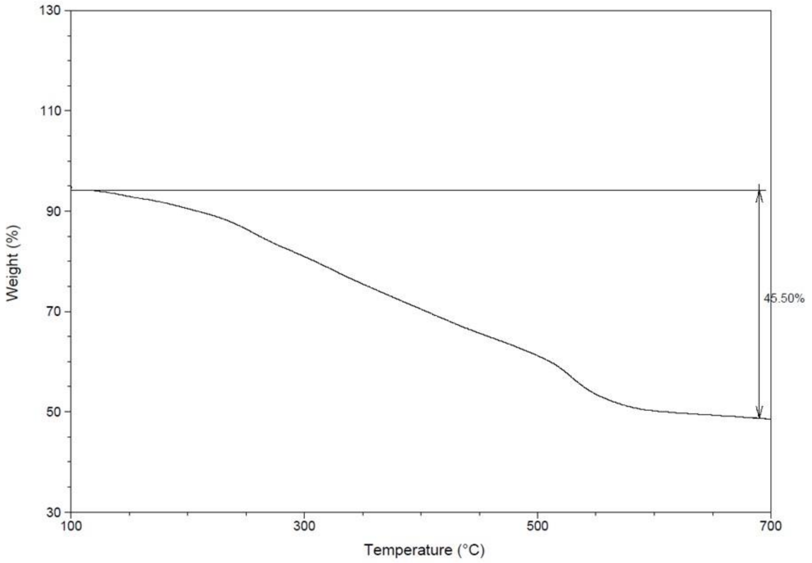

Supplementary Figure 2: TGA analysis of **1**@AuNP

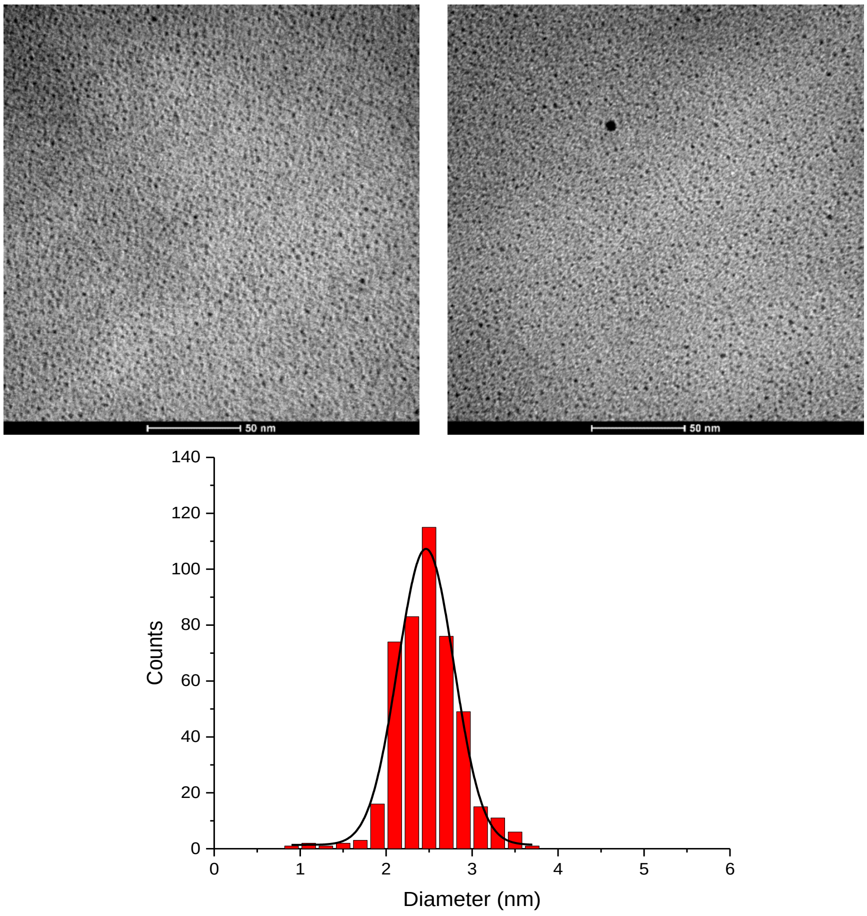

Supplementary Figure 3: TEM image, size distribution of **1**@AuNP and fitting curve parameters (average diameter = 2.4 nm, σ = 0.6 nm)

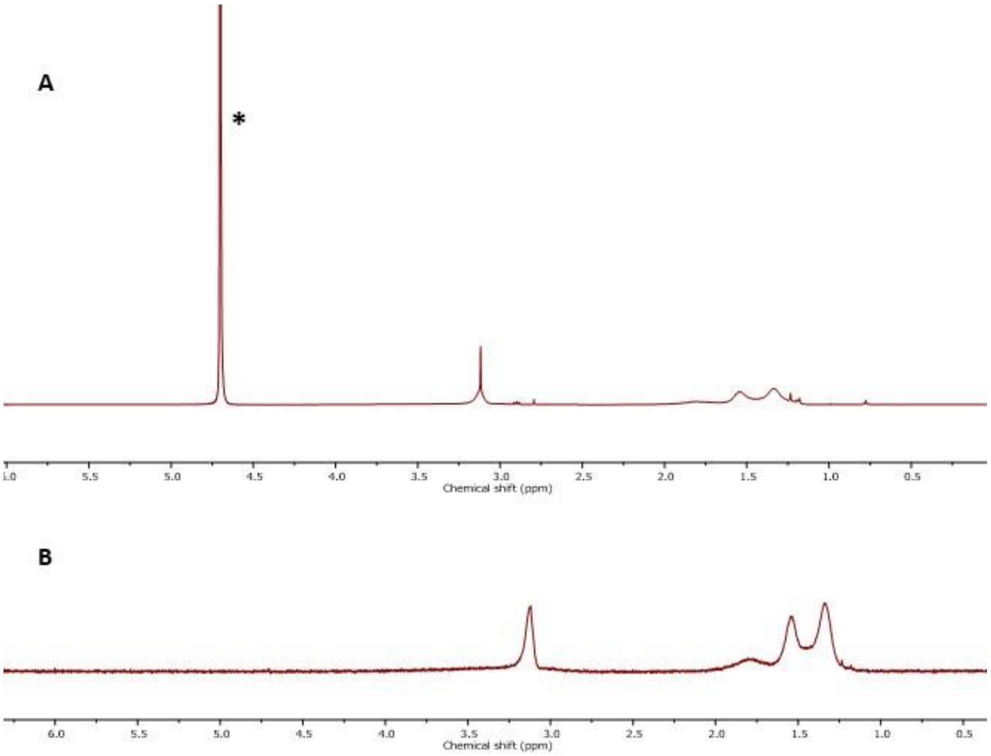

Supplementary Figure 4: A) ^{1}HNMR spectrum of **1**@AuNP in D_2O. B) Diffusion filter NMR spectrum of **1**@AuNP in D_2O. *: residual solvent signals

1.4.2. 2@AuNP

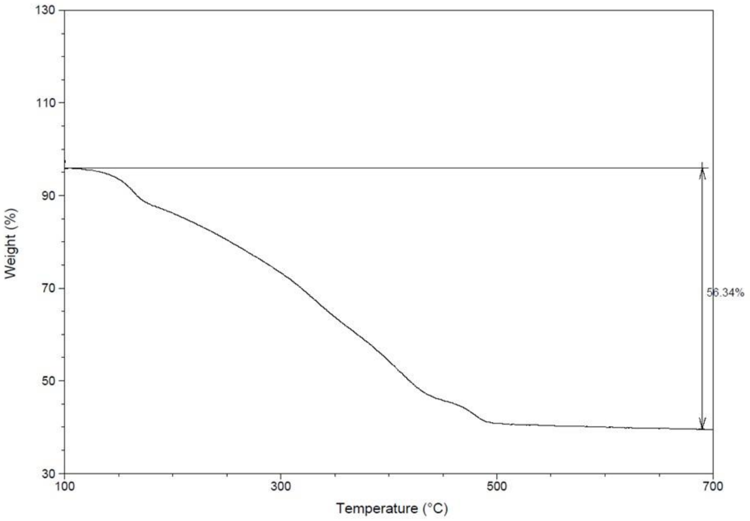

Supplementary Figure 5: TGA analysis of **2**@AuNP

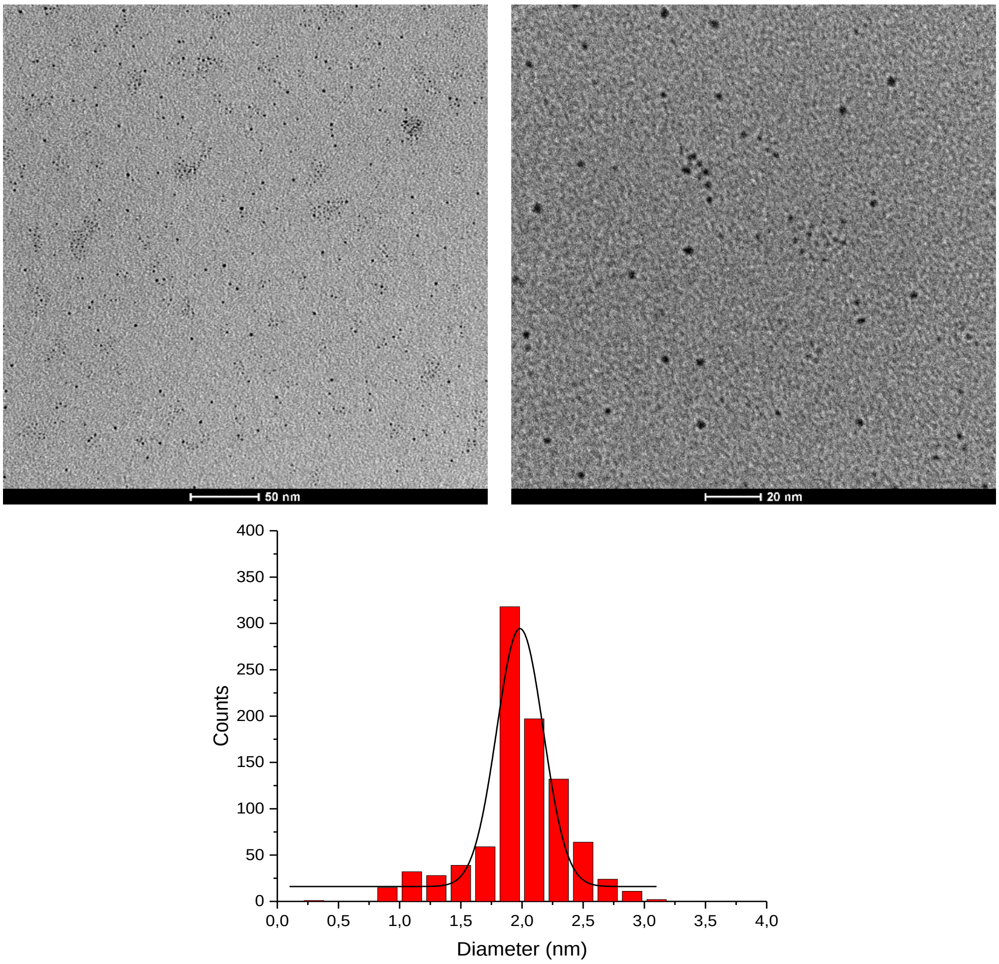

Supplementary Figure 6: TEM image, size distribution of **2**@AuNP and fitting curve parameters (average diameter = 1.9 nm, σ = 0.4 nm)

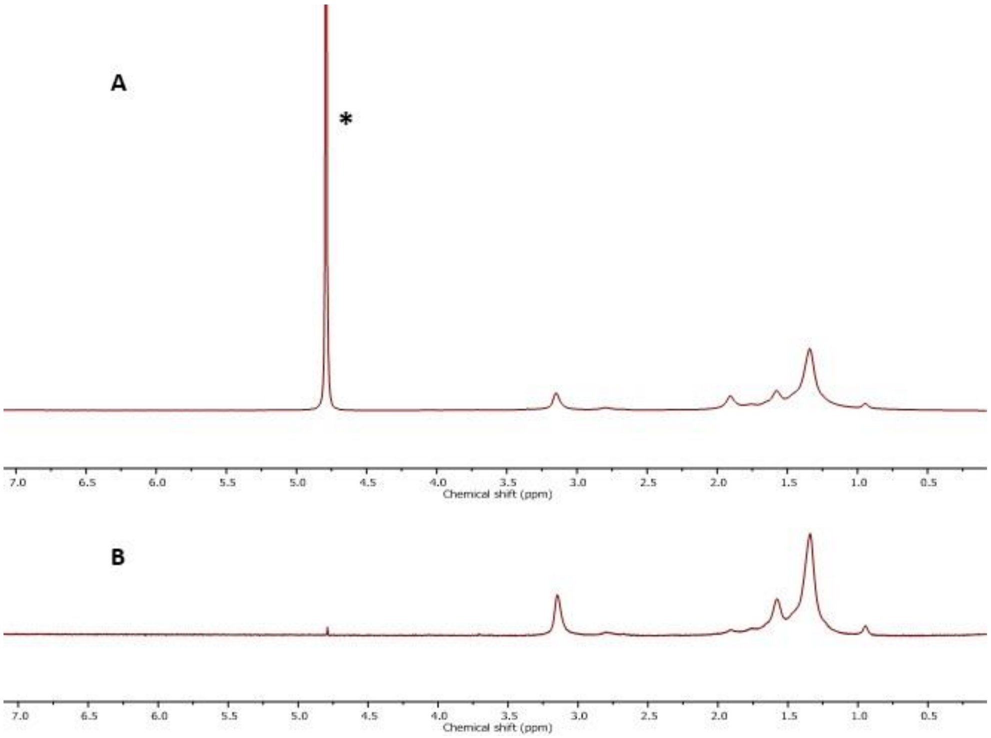

Supplementary Figure 7: A) ^{1}HNMR spectrum of **2**@AuNP in D_2O. B) Diffusion filter NMR spectrum of **2**@AuNP in D_2O. *: residual solvent signals

1.4.3. 3@AuNP

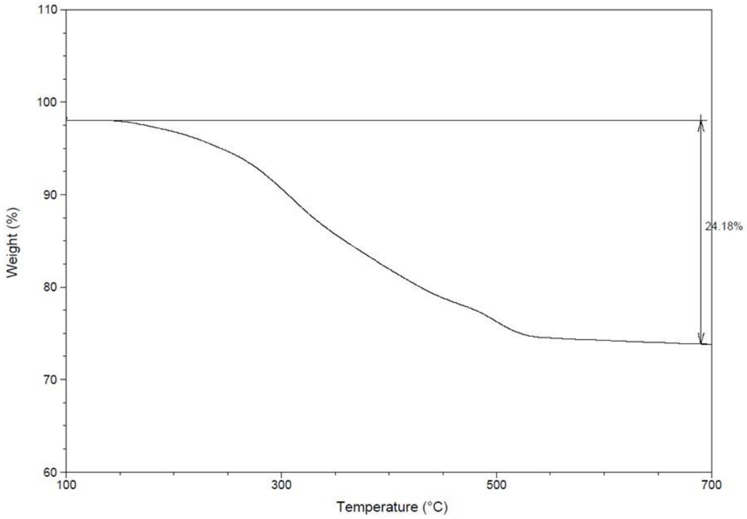

Supplementary Figure 8: TGA analysis of **3**@AuNP

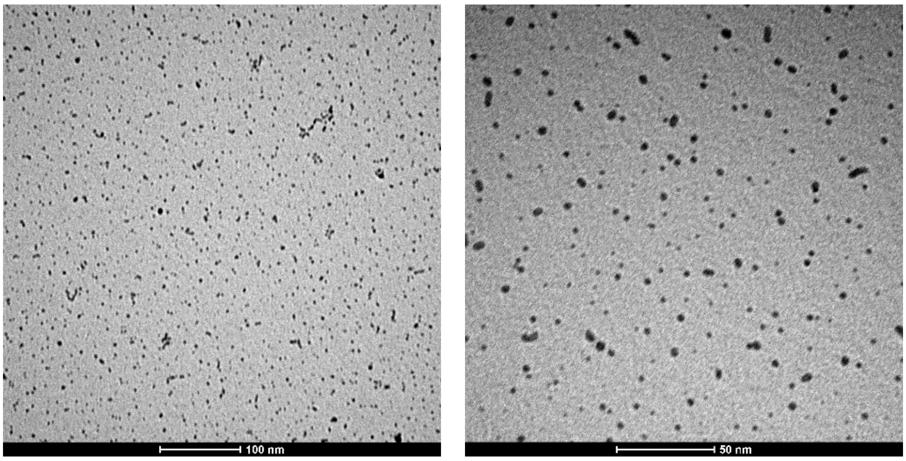

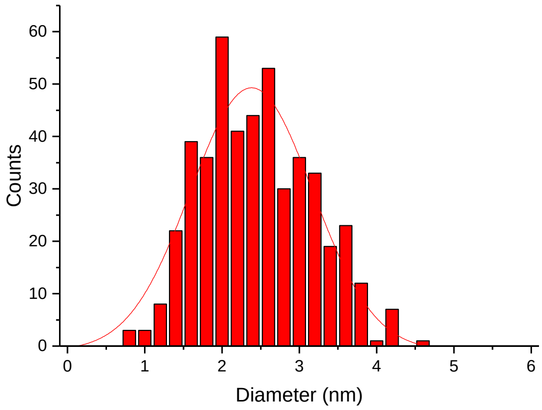

Supplementary Figure S9: TEM image, size distribution of **3**@AuNP and fitting curve parameters (average diameter = 2.4 nm, σ = 0.8 nm)

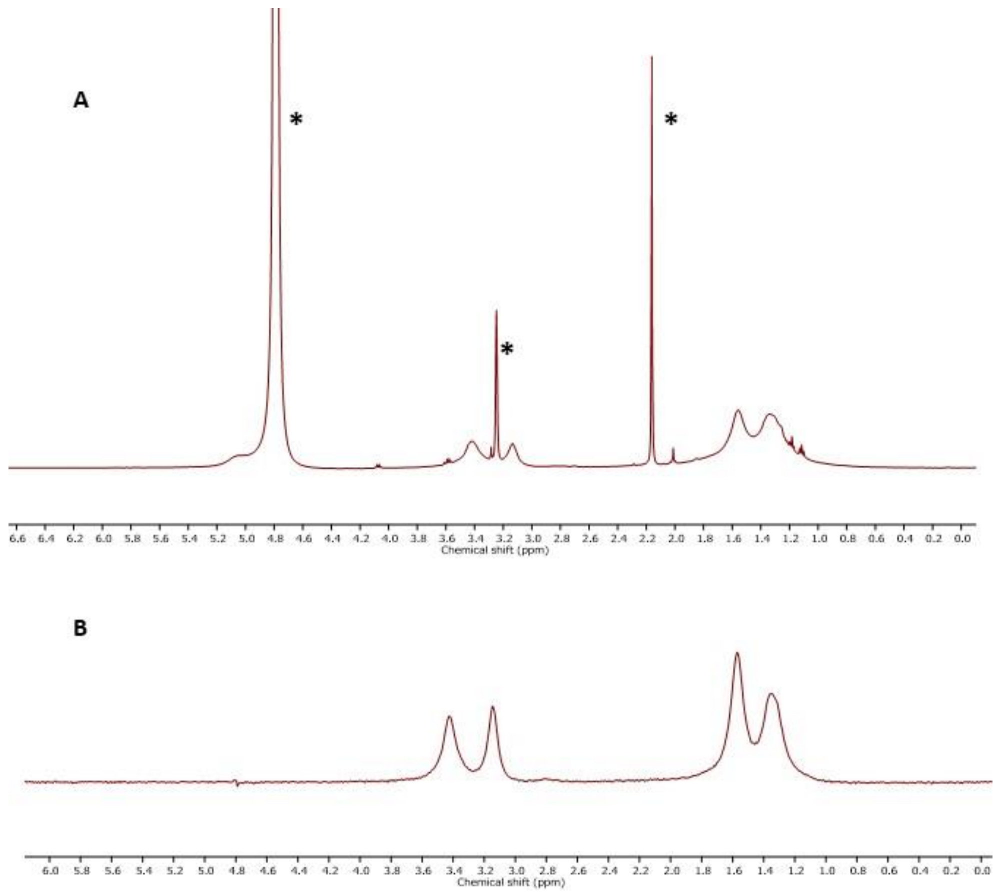

Supplementary Figure 10: A) ^{1}HNMR spectrum of **3**@AuNP in D_2O. B) Diffusion filter NMR spectrum of **3**@AuNP in D_2O. *: residual solvent signals

1.4.4. 6@AuNP

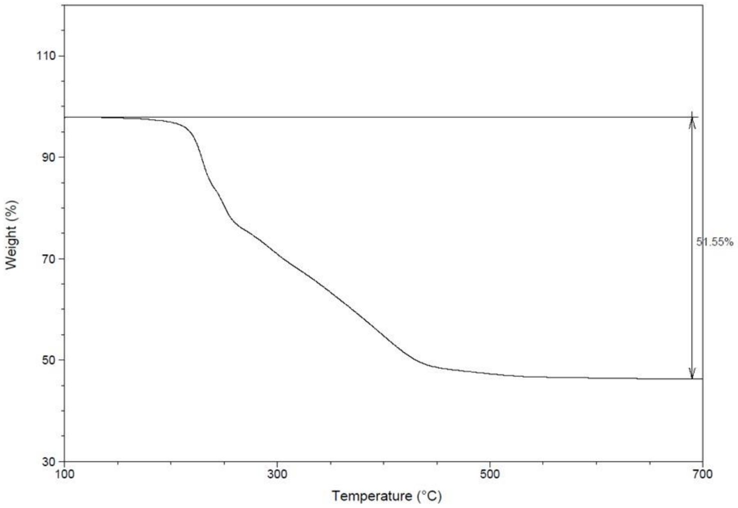

Supplementary Figure 11: TGA analysis of **6**@AuNP

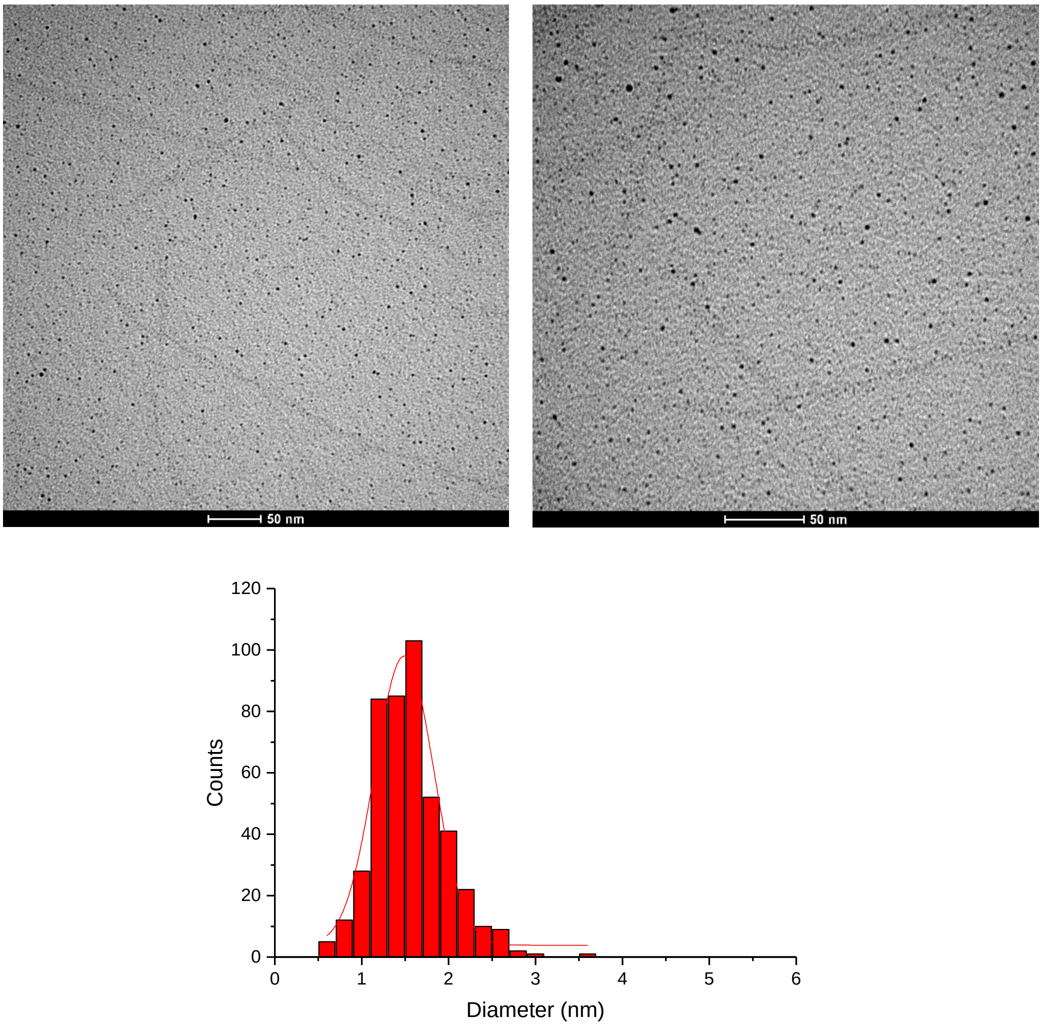

Supplementary Figure 12: TEM image, size distribution of **6**@AuNP and fitting curve parameters (average diameter = 1.5 nm, σ = 0.3 nm)

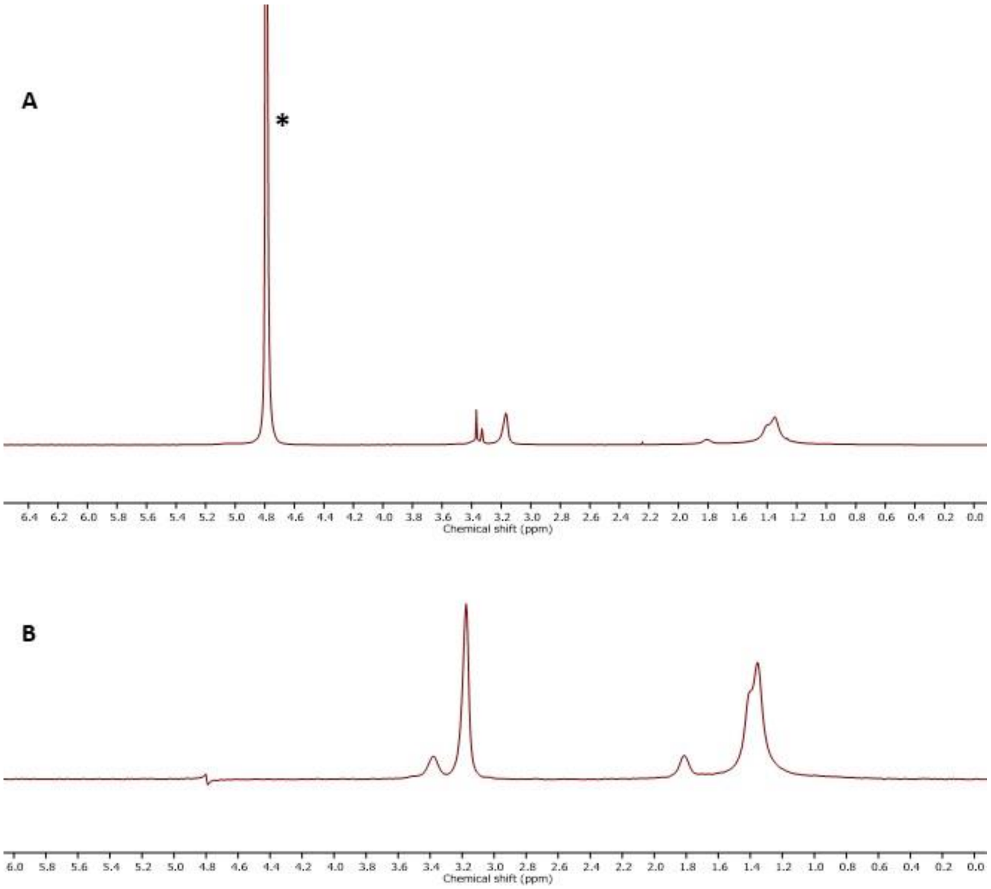

Supplementary Figure 13: A) ^{1}HNMR spectrum of **6**@AuNP in D_2O. B) Diffusion filter NMR spectrum of **6**@AuNP in D_2O. *: residual solvent signals

1.4.5. 7@AuNP

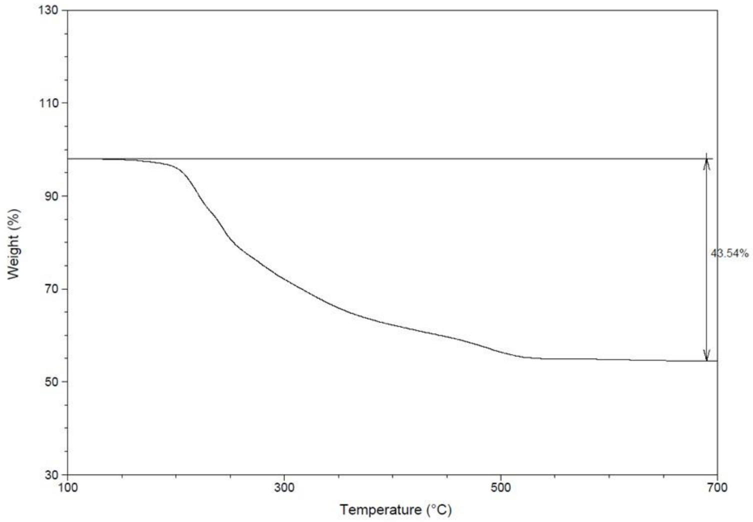

Supplementary Figure 14: TGA analysis of **7**@AuNP

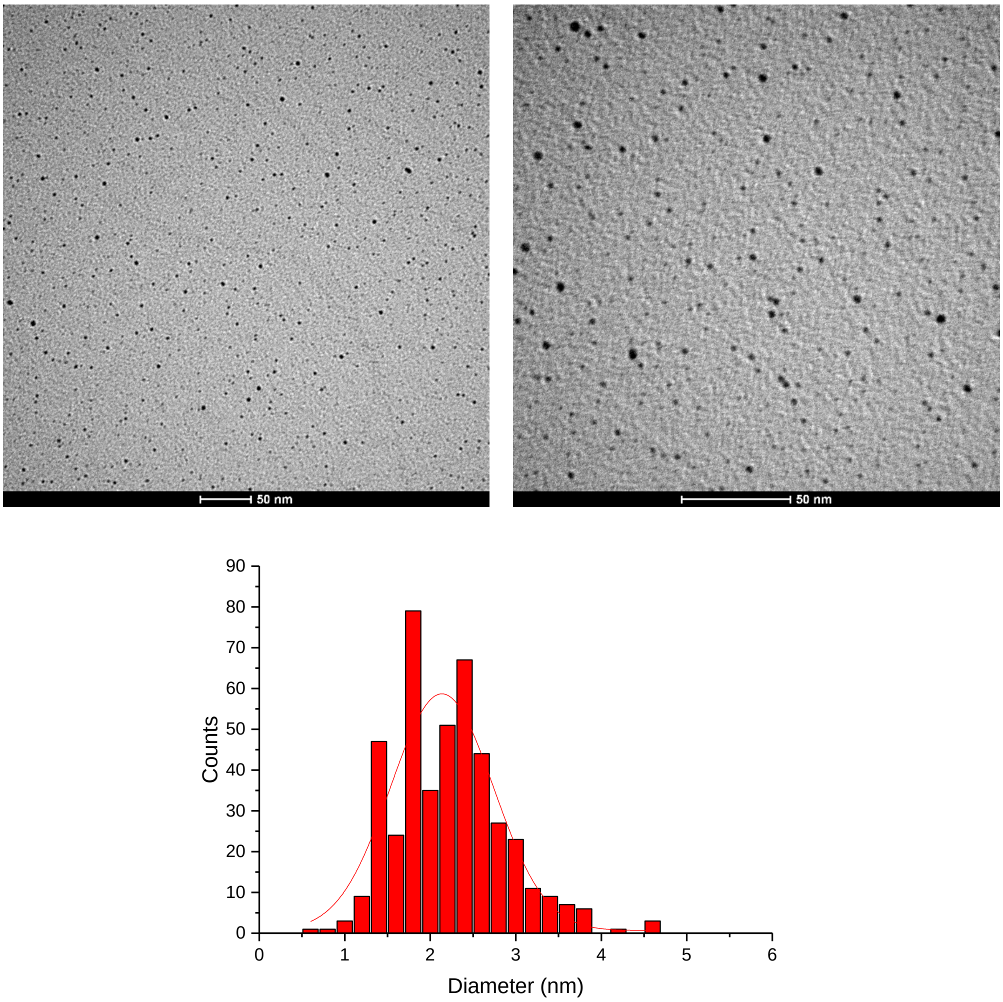

Supplementary Figure 15: TEM image, size distribution of **7**@AuNP and fitting curve parameters (average diameter = 2.1 nm, σ = 0.6 nm)

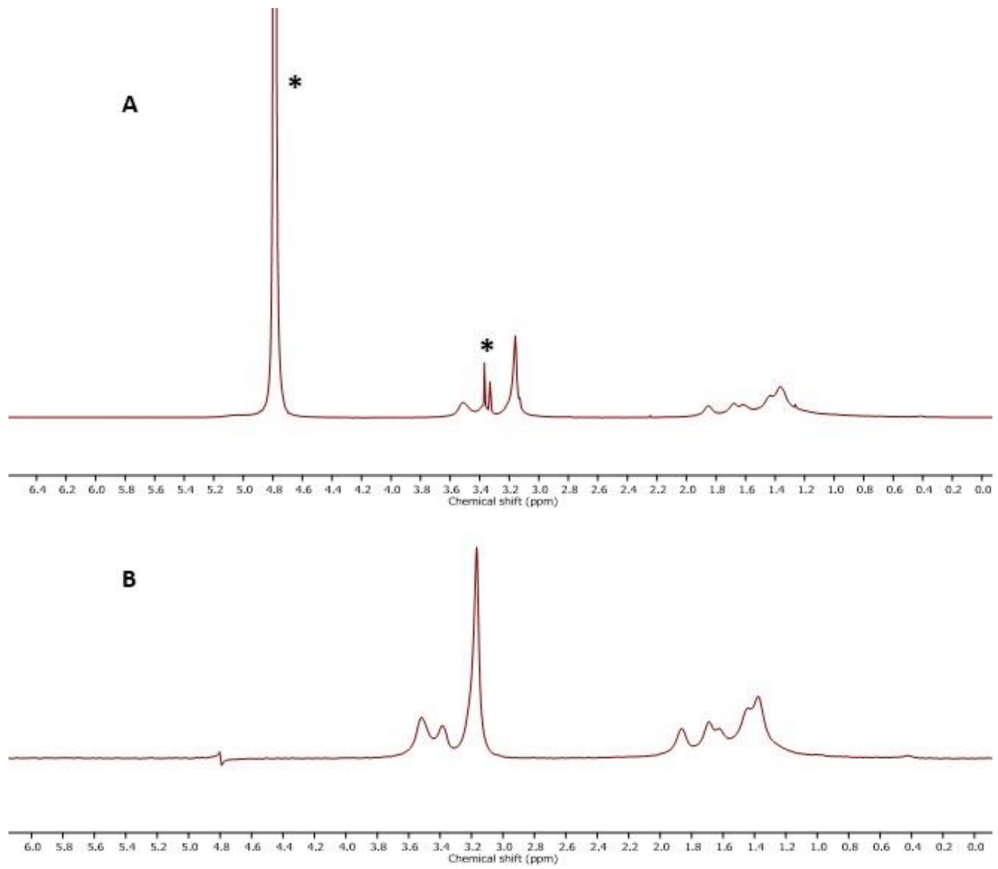

Supplementary Figure 16: A) ^{1}HNMR spectrum of **7**@AuNP in D_2O. B) Diffusion filter NMR spectrum of **7**@AuNP in D_2O. *: residual solvent signals

1.4.6. AuNPs Z-potential and summary characterization table

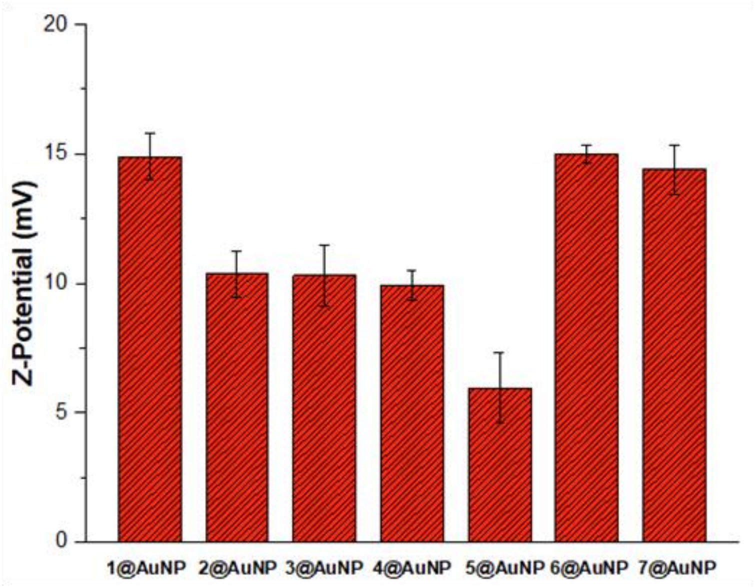

Supplementary Figure 17: Z-potential of **1-7**@AuNPs in PBS 10 mM, pH 7, 25 ºC. Error bars were calculated by propagation.

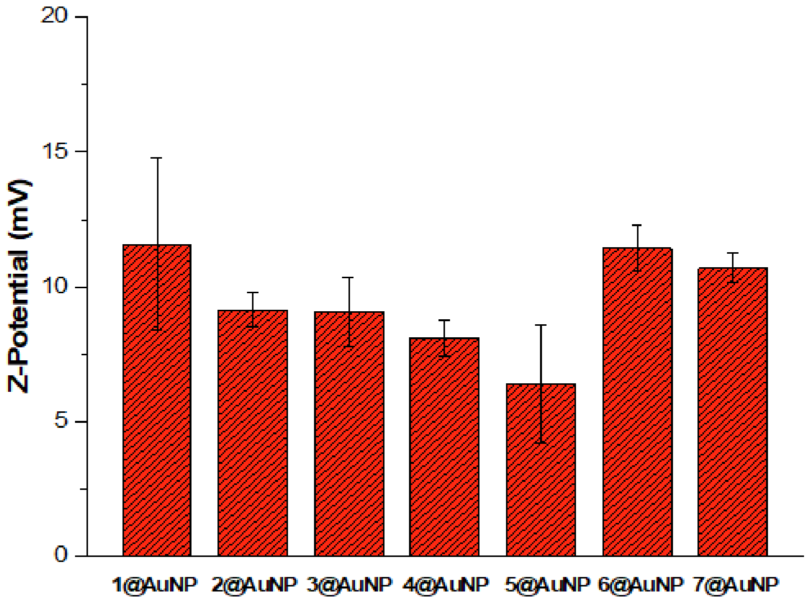

Supplementary Figure 18: Z-potential of **1-7**@AuNPs in HEPES 10 mM, NaCl 100 mM, pH 7, 25 ºC. Error bars were calculated by propagation.

Supplementary Table 1: Summary table of AuNP characterizations.[a]

Entry	Core size (nm)[b]	Hydrodynamic size (nm)[c]	Z-potential (mV)	Number of ligand per NP	Number of gold per NP	Footprint (nm^2)[d]
**1**@AuNP	2.4±0.6	9.6±0.5	11.6±3.2	260±13	427±64	0.07±0.04
**1'**@AuNP	1.4±0.2[e]	8.4±0.7	13.5±1.3	47±2.4[e]	90±14[e]	0.15±0.04[e]
**2**@AuNP	1.9±0.4	7.2±0.5	9.1±1.3	73±4	427±64	0.25±0.17
**3**@AuNP	2.4±0.8[e]	8.1±0.6	8.1±0.7	66±3[e]	127±19[e]	0.12±0.05[e]
**4**@AuNP	1.6±0.3[e]	5.1±0.4	6.4±2.2	44±2[e]	127±19[e]	0.18±0.07[e]
**5**@AuNP	1.6±0.3	10.4±0.6	11.4±0.9	53±4	104±16	0.13±0.03
**6**@AuNP	1.5±0.3	7.4±0.4	10.7±0.5	106±6	286±43	0.13±0.08
**7**@AuNP	2.1±0.6	10.7±0.7	9.1±1.3	73±4	427±64	0.25±0.17

a) errors are the standard deviation from triplicate experiments or errors propagation; b) From TEM analysis; c) measured at 25 °C in 10 mM HEPES buffer, pH 7.0, containing 100 mM NaCl; d) Average surface area occupied by each ligand; e) data from ref. 2.

1.5. Preparation of neutrally charged fluorogenic liposomes

Fluorogenic liposomes with calcein. 7.5 mg of phosphatidylcholine was dried for 4h in a vacuum pump and then hydrated with buffer solution of the fluorophore (1 ml, calcein 50 mM, HEPES 10 mM, NaCl 100 mM, pH 7) at 42ºC for 40 minutes. Then, 6 cycles of freeze/thaw were performed, followed by a 15 times extrusion filtration with a polycarbonate membrane (0.1 µm, 19 mm). Size extrusion chromatography (G75) with buffer solution (HEPES 10 mM, NaCl 100 mM, pH 7) was used to remove extravesicular fluorophore. The liposome samples were stored at 4ºC.

Fluorogenic liposomes with nile red. 7.5 mg of phosphatidylcholine and nile red solution in THF (98.9 µl) were dried for 4h in a vacuum pump and then hydrated with buffer solution (1 ml, HEPES 10 mM, pH 7) at 42ºC for 40 minutes. Then, 6 cycles of freeze/thaw were performed, followed by a 15 times extrusion filtration with a polycarbonate membrane (0.1 µm, 19 mm). Size extrusion chromatography (G75) with buffer solution (HEPES 10 mM, pH 7) was used to remove extravesicular fluorophore. The liposome samples were stored at 4ºC.

Fluorogenic liposomes with calcein. 7.5 mg of phosphatidylcholine was dried for 4h in a vacuum pump and then hydrated with buffer solution of the fluorophore (1 ml, calcein 50 mM, HEPES 10 mM, glucose 200 mM, pH 7) at 42ºC for 40 minutes. Then, 6 cycles of freeze/thaw were performed, followed by a 15 times extrusion filtration with a polycarbonate membrane (0.1 µm, 19 mm). Size extrusion chromatography (G75) with buffer solution (HEPES 10 mM, glucose 200 mM, pH 7) was used to remove extravesicular fluorophore. The liposome samples were stored at 4ºC.

1.6. Preparation of negatively charged fluorogenic liposomes

Fluorogenic liposomes with calcein. 7.5 mg of phosphatidylcholine and phosphatidylglycerol solution in $CHCl_3$ (20 µl) were dried for 4h in a vacuum pump and then hydrated with buffer solution of the fluorophore (1 ml, calcein 50 mM, HEPES 10 mM, NaCl 100 mM, pH 7) at 42ºC for 40 minutes. Then, 6 cycles of freeze/thaw were performed, followed by a 15 times extrusion filtration with a polycarbonate membrane (0.1 µm, 19 mm). Size extrusion chromatography (G75) was used to remove extravesicular fluorophore. The liposome was stored at 4ºC.

Fluorogenic liposomes with nile red. 7.5 mg of phosphatidylcholine and phosphatidylglycerol solution in $CHCl_3$ (20 µl) were dried with nile red solution in THF 898.9 ul) for 4h in a vacuum pump and then hydrated with buffer solution (1 ml, 50 mM, HEPES, pH 7) at 42ºC for 40 minutes. Then, 6 cycles of freeze/thaw were performed, followed by a 15 times extrusion filtration with a polycarbonate membrane (0.1 µm, 19 mm). Size extrusion chromatography (G75) was used to remove extravesicular fluorophore. The liposome samples were stored at 4ºC.

Fluorogenic liposomes with calcein. 7.5 mg of phosphatidylcholine and phosphatidylglycerol solution in $CHCl_3$ (20 µl) were dried for 4h in a vacuum pump and then hydrated with buffer solution of the fluorophore (1 ml, calcein 50 mM, HEPES 10 mM, glucose 200 mM, pH 7) at 42ºC for 40 minutes. Then, 6 cycles of freeze/thaw were performed, followed by a 15 times extrusion filtration with a polycarbonate membrane (0.1 µm, 19 mm). Size extrusion chromatography (G75) with buffer solution (HEPES 10 mM, glucose 200 mM, pH 7) was used to remove extravesicular fluorophore. The liposome samples were stored at 4ºC.

1.7. Preparation of rigidified fluorogenic liposomes

Fluorogenic liposomes with calcein. 7.5 mg of phosphatidylcholine and cholesterol solution in CHCl3 (35 µl) were dried for 4h in a vacuum pump and then hydrated with buffer solution of the fluorophore (1 ml, calcein 50 mM, HEPES 10 mM, NaCl 100 mM, pH 7) at 42ºC for 40 minutes. Then, 6 cycles of freeze/thaw were performed, followed by a 15 times extrusion filtration with a polycarbonate membrane (0.1 µm, 19 mm). Size extrusion chromatography (G75) was used to remove extravesicular fluorophore. The liposome samples were stored at 4ºC.

1.8. Characterization of the fluorogenic liposomes

Supplementary Table 2: Summary table of hydrodynamic diameter (HEPES 10 mM, NaCl 100 mM, pH 7, 25 ºC) and Z-potential (PBS 10 mM, pH 7, 25 ºC) values for the prepared liposomes.

Entry	Liposome	Size (nm)	PDI	Z-potential (mV)
1	PC-calcein-liposome	79.30	0.15	-1.74
2	PC+PG-calcein-liposome	98.47	0.06	-7.69
3	PC+cholesterol-calcein-liposome	96.62	0.14	-0.69
4	PC-nile_red-liposome	96.98	0.14	-1.62
5	PC+PG-nile_red-liposome	95.63	0.07	-5.76

1.9. Liposome experiments

Fluorescence recovery experiments were initiated by the addition of a nanoparticles stock solution to 2 ml buffered solution (HEPES 10 mM, NaCl 100 mM, pH 7.0 or HEPES 10 mM, glucose 200 mM, pH 7.0) containing liposomes (22 μM phospholipid concentration) in a quartz cell. Sample emission at 25 ºC was measured followed until no further variation were detected (usually within 5 minutes). The maximum fluorophore emission was measured after addition of Triton X100 in the cell.

2. CryoEM liposome characterization

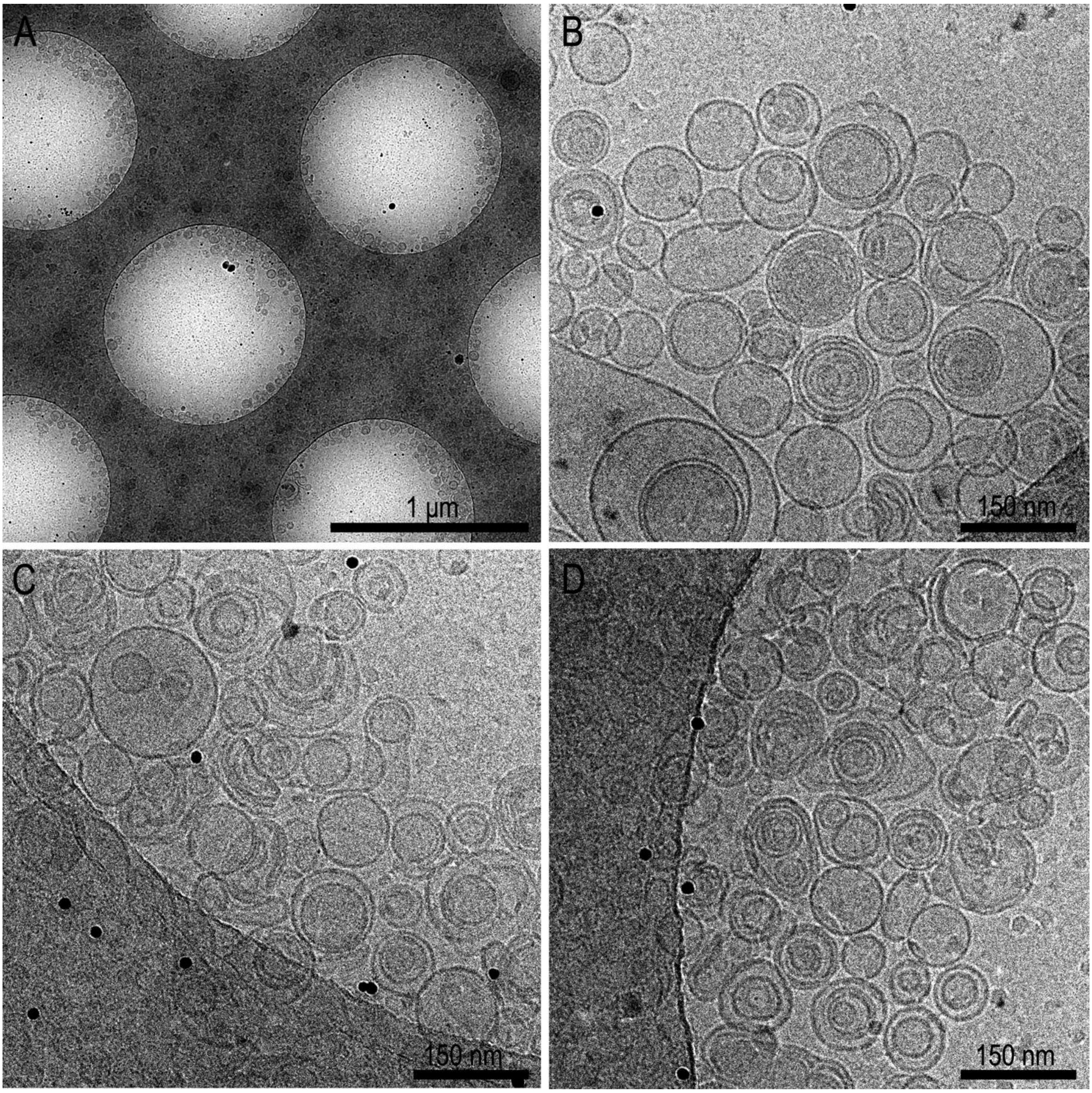

Supplementary Figure 19. Cryo-EM of PC liposomes: cryo-EM projection images showing the liposomes, used to calculate the size distribution reported in figure 5 (black bars). Black dots visible are spurious impurities.

3. Additional experiments on calcein loaded liposomes

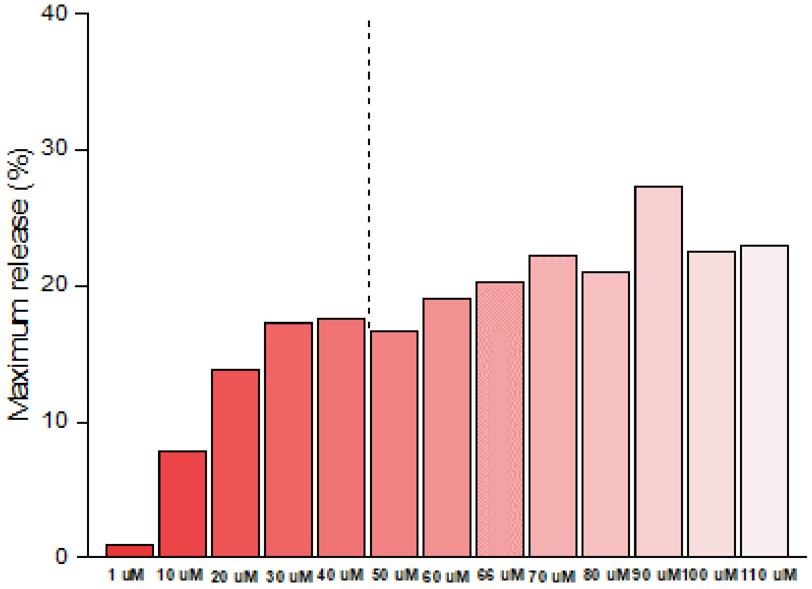

Supplementary Figure 20: Maximum release of calcein after incubation with increasing concentrations 1 µM-110 µM of **1**@AuNP (HEPES 10 mM, NaCl 100 mM, pH 7.0, [PC] = 22 µM, 25 ºC). Dashed line indicates the estimated saturation concentration.

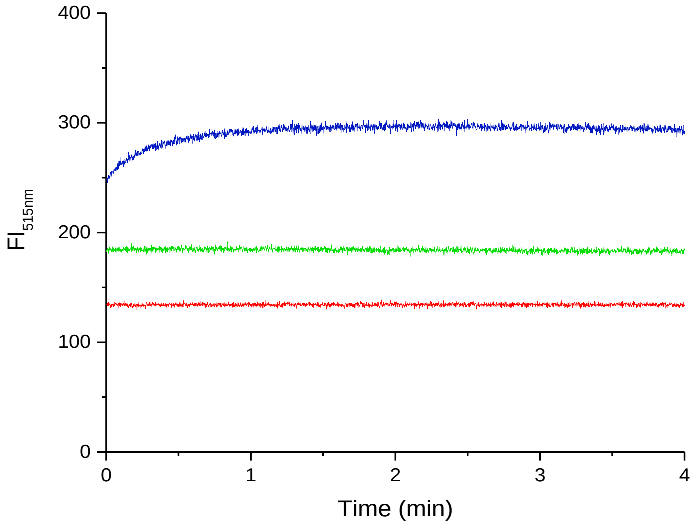

Supplementary Figure 21: Time-dependent emission of neutrally charged liposome alone (red), after addition of **4**@AuNP (green), after addition of **1**@AuNP (blue), (HEPES 10 mM, NaCl 100 mM, pH 7, PC 22 µM, NPs-thiols 66 µM, 25 ºC).

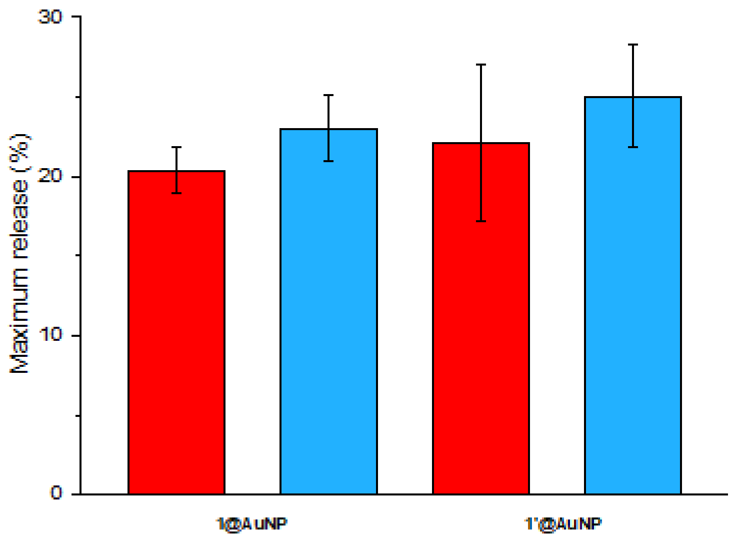

Supplementary Figure 22: Maximum release of calcein from PC (red) and PC/PG (blue) liposomes after addition of 1 or 1'@AuNPs, which feature the same coating and different average sizes (see Table 1 and Table S1). Experimental conditions: [HEPES] = 10 mM, [NaCl] = 100 mM, [PC]/ [PC+PG]/ [PC+chol] = 22 µM, [AuNPs-thiols] = 66 µM, pH 7.0, 25 °**C**. Error bars were calculated by propagation.

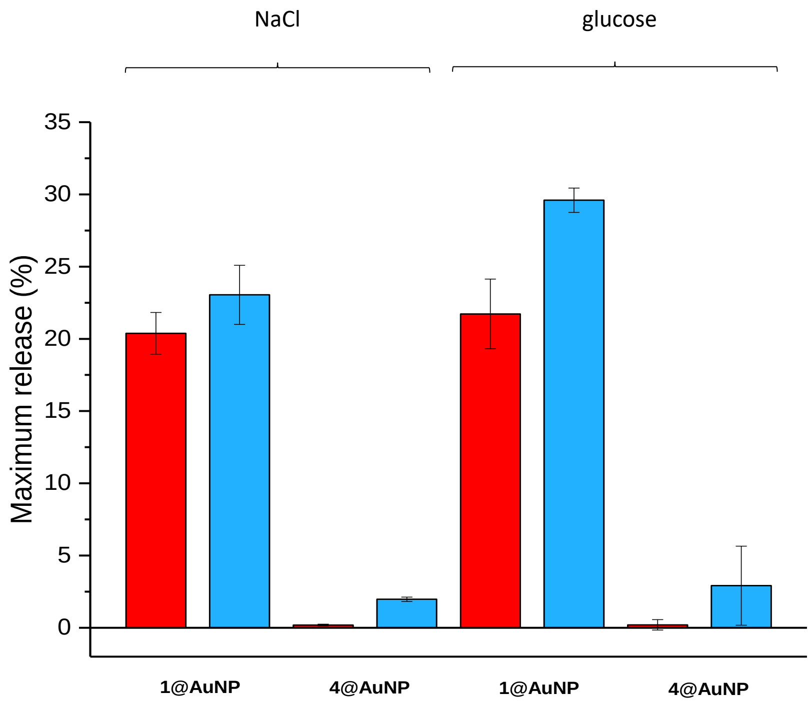

Supplementary Figure 23: Maximum release of calcein from PC (red) and PC/PG (blue) liposomes after addition of 1 or 4@AuNPs, with different buffer solutions. Experimental conditions: [HEPES] = 10 mM, [NaCl] = 100 mM or [Glucose] = 200 mM, [PC]/ [PC+PG]/ [PC+chol] = 22 µM, [AuNPs-thiols] = 66 µM, pH 7.0, 25 °**C**. Error bars were calculated by propagation.

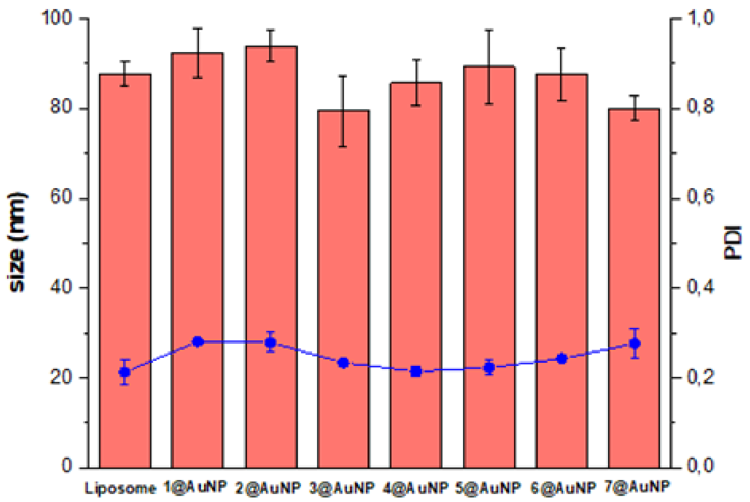

Supplementary Figure 24: Hydrodynamic size (red bars) and PDI (blue spots) of PC liposomes and after the addition of **1-7**@AuNPs. Experimental conditions: [HEPES] = 10 mM, [NaCl] = 100 mM, pH 7.0, [PC] = 22 µM, 25ºC. Error bars were calculated by propagation.

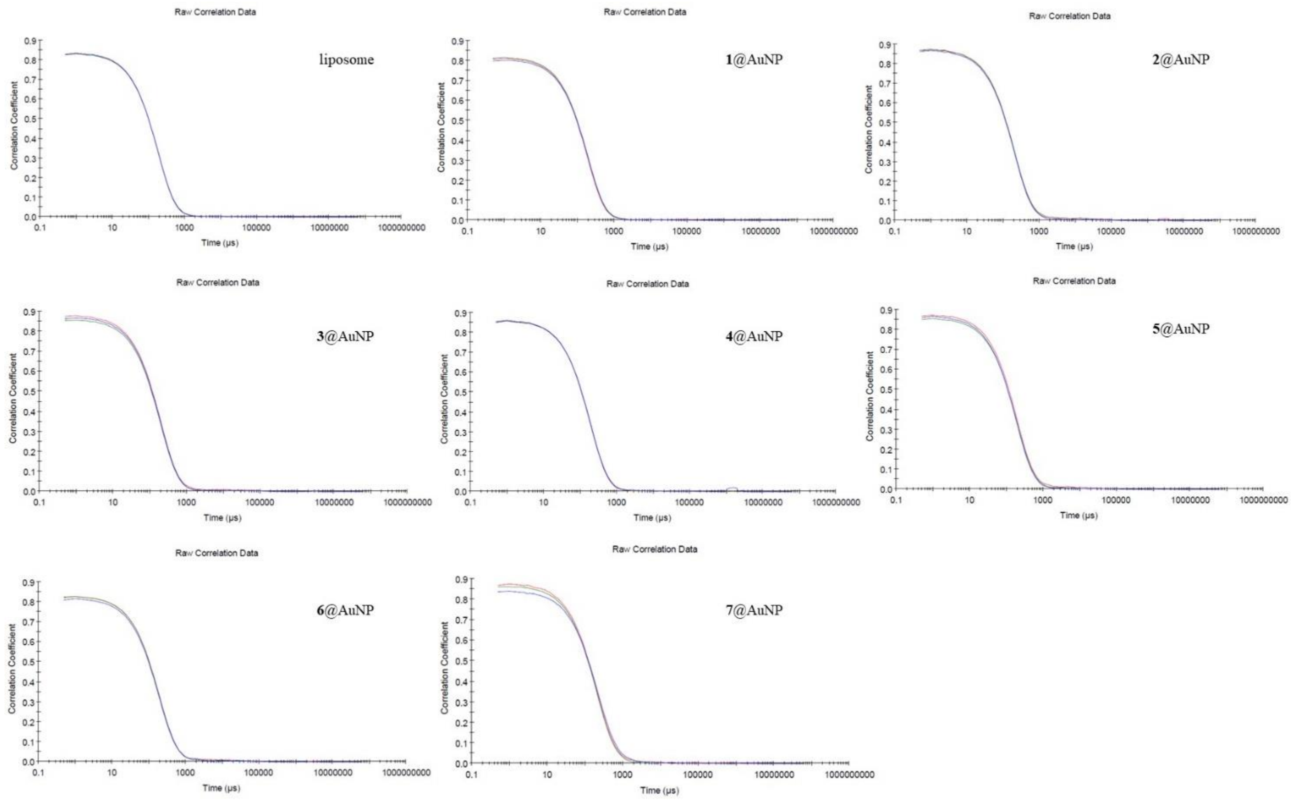

Supplementary Figure 25: Correlation function of PC liposomes and after the addition of **1-7**@AuNPs. Experimental conditions: [HEPES] = 10 mM, [NaCl] = 100 mM, pH 7.0, [PC] = 22 µM, 25ºC.

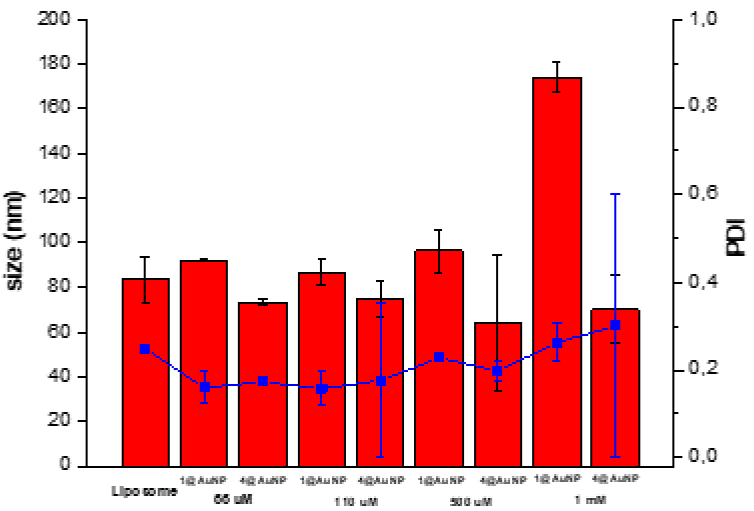

Supplementary Figure 26: Hydrodynamic size (red bars) and PDI (blue spots) of PC liposomes and after the addition of **1**@AuNP and **4**@AuNP. Experimental conditions: [HEPES] = 10 mM, [NaCl] = 100 mM, pH 7.0, [PC] = 22 µM, 25ºC. Error bars were calculated by propagation.

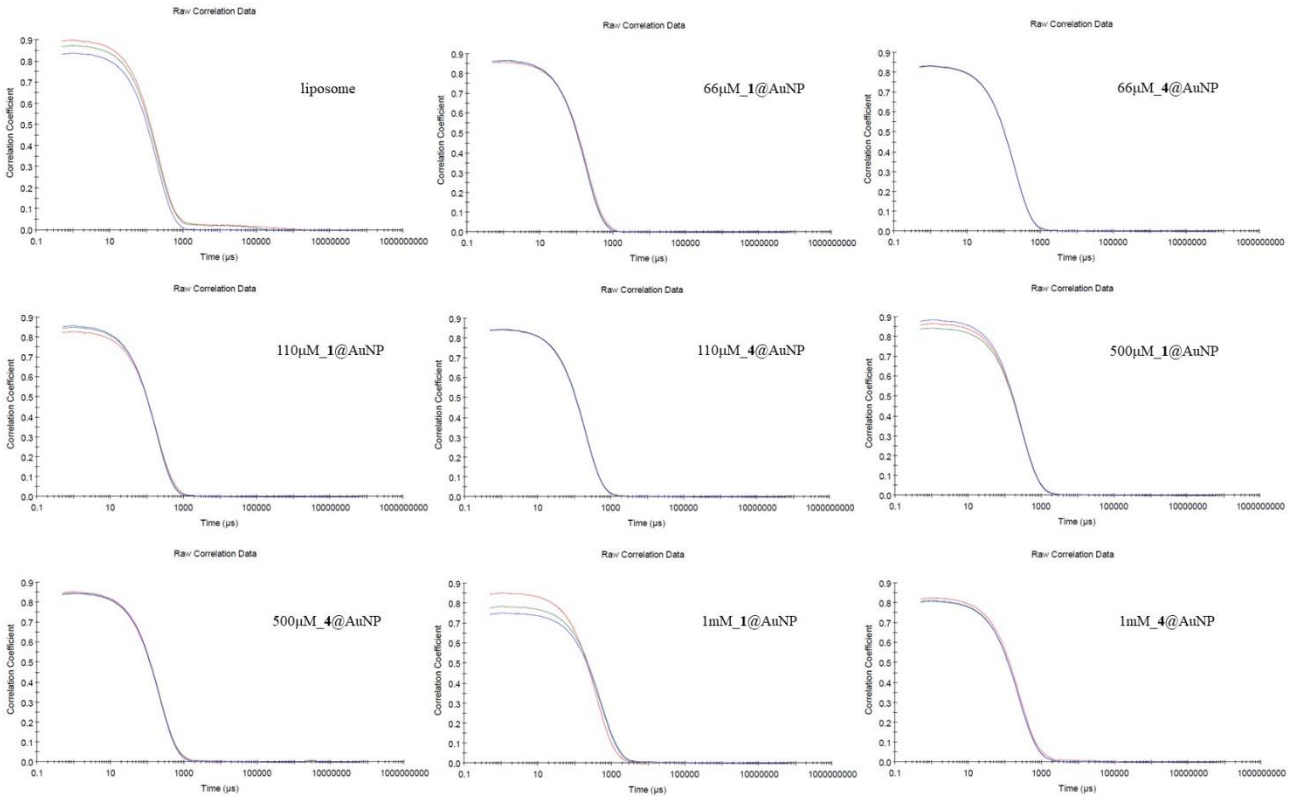

Supplementary Figure 27: Correlation function of PC liposomes and after the addition of **1**@AuNP and **4**@AuNP. Experimental conditions: [HEPES] = 10 mM, [NaCl] = 100 mM, pH 7.0, [PC] = 22 µM, 25ºC.

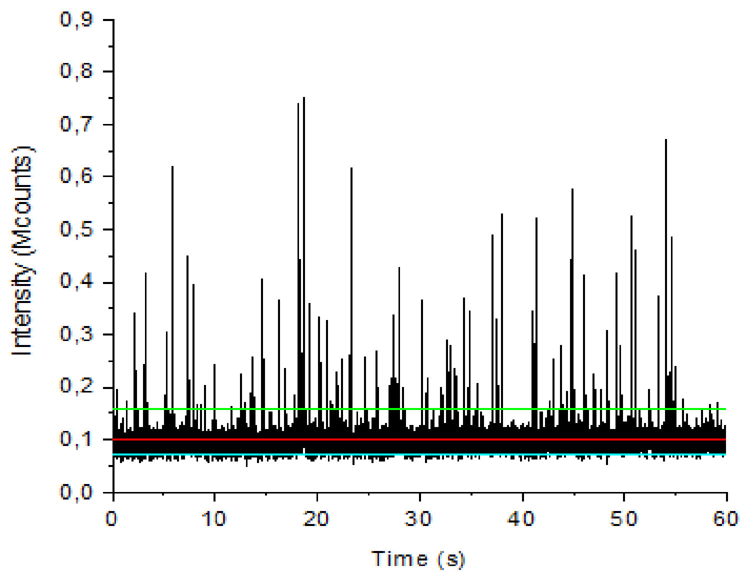

Supplementary Figure 28: Time dependent emission intensity of PC charged liposomes (black) and intensity of the threshold set: 0.07 Mcounts (cyan), 0.10 Mcounts (red) and 0.16 Mcounts (green).

Supplementary Table 3: Decay life-times and relative amplitude for different threshold.

Entry	Threshold (Mcounts)	τ_1 (ns)	τ_2 (ns)	A_1 (%)	A_2 (%)
1	0.07	4	0.290	45.7	54.3
2	0.08	4	0.287	44.5	55.5
3	0.09	4	0.285	41.0	59.0
4	0.1	4	0.274	32.5	67.5
5	0.11	4	0.272	21.8	78.2
6	0.12	4	0.274	14.2	85.8
7	0.13	4	0.280	10.9	89.1
8	0.135	4	0.283	10.1	89.9
9	0.14	4	0.280	9.7	90.4
10	0.15	4	0.283	8.9	91.2
11	0.16	4	0.293	8.2	91.8

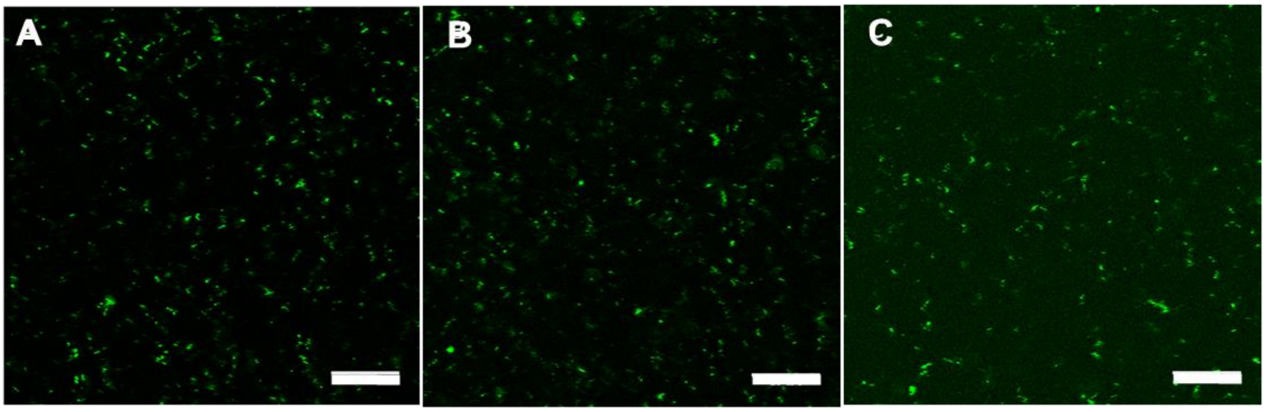

Supplementary Figure 29. Confocal microscopy images of A) calcein-loaded PC liposomes, B) same sample as A after the addition of **4**@AuNPs, C) same sample as A after the addition of **1**@AuNPs. Scale bar: 10 μm. Experimental conditions: [HEPES] = 10 mM, [NaCl] = 100 mM, [PC] = 22 μM, [AuNPs-thiols] = 66 μM, pH 7.0, 25 ºC, λ_{exc} = 488 nm, λ_{em} > 510 nm.

The release of calcein from liposomes was monitored with fluorescence confocal microscopy. Images recorded from samples of calcein-loaded PC liposomes showed the presence of scattered green dots, at the resolution of the microscope, these correspond to the residual emission of individual or small groups of liposomes (Figure S29A). The addition of **4**@AuNP (Figure S29B) did not produce any significant changes in this pattern, consistent with the results of the previous experiments. However, for **1**@AuNP (Figure S29C), a relevant increase in the green background emission was clearly detected, while the dotted pattern of the emission from the liposomes remained substantially unaltered. We ascribed this effect to the release of calcein into the bulk solution. According to confocal images, this occurred without affecting the integrity of the liposomes.

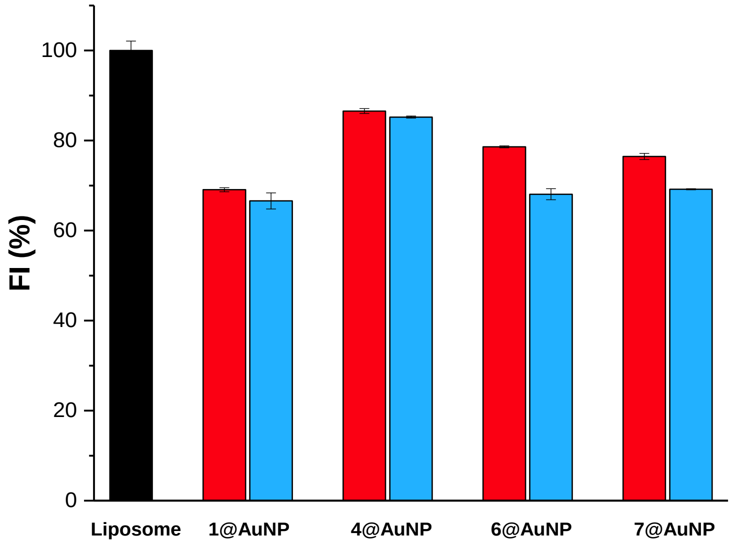

Supplementary Figure 30: Relative fluorescence intensity of nile-red-loaded PC alone (black, set to 100%), PC (red) and PC/PG liposomes (blue) after the addition of **1**, **4**, **6**, and **7**@AuNPs. Experimental conditions: [HEPES] = 10 mM, [NaCl] = 100 mM, [PC]/ [PC+PG] = 22 µM, , [AuNPs-thiols] = 66 µM, pH 7.0, 25 °C, λ_{exc} = 534 nm, λ_{em} = 629 nm. Error bars were calculated by propagation.

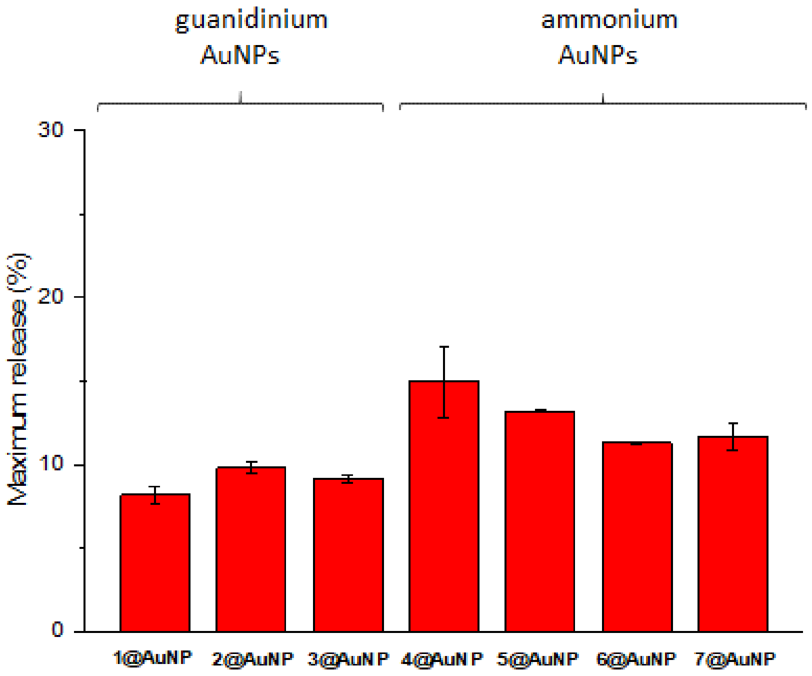

Supplementary Figure 31: Maximum release of calcein from PC (red) liposomes after addition of **1-7**@AuNPs in the presence of dimethylphosphate. Experimental conditions: [PBS] = 10 mM, [dimethyl phosphate] = 10 mM, [NaCl] = 90 mM, [PC] = 22 µM, [AuNPs-thiols] = 66 µM, pH 7.0, 25 ºC. Error bars were calculated by propagation.

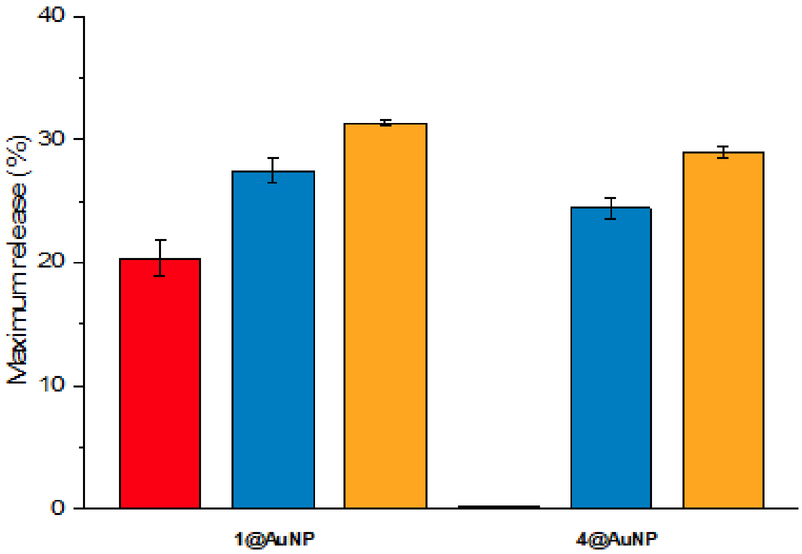

Supplementary Figure 32: Maximum release of calcein from PC liposomes after addition of **1**@AuNP and **4**@AuNP at 25ºC (red) 30ºC (blue) and 40ºC (orange). Experimental conditions: [HEPES] = 10 mM, [NaCl] = 100 mM, [PC] = 22 µM, [AuNPs-thiols] = 66 µM, pH 7.0, 25 ºC, λ_{exc} = 534 nm, λ_{em} = 629 nm. Error bars were calculated by propagation.

4. Molecular dynamics simulations

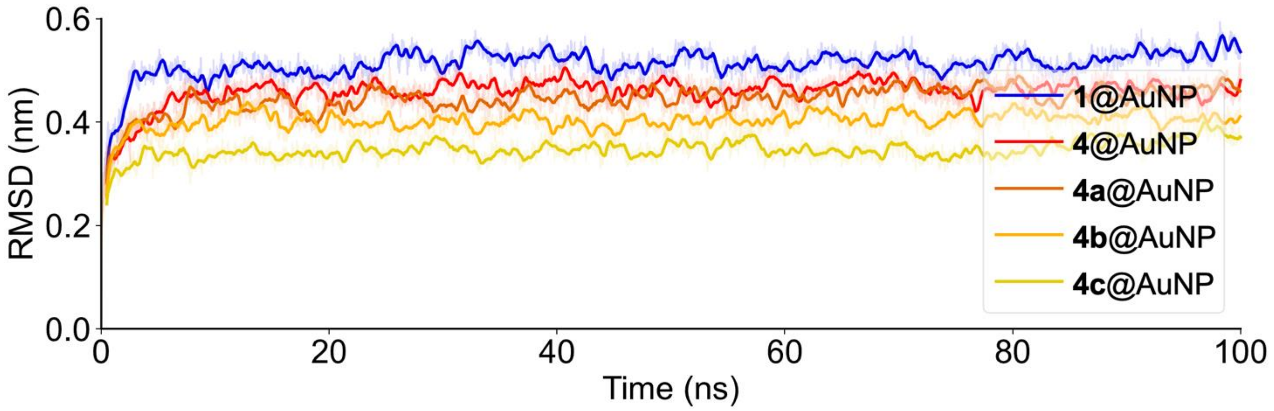

Supplementary Figure 33: Root-mean-squared deviation (RMSD) of the atomic coordinates for the AuNPs. All the structures converge within the first 5 ns of simulation as evidenced by the flattening of the curve.

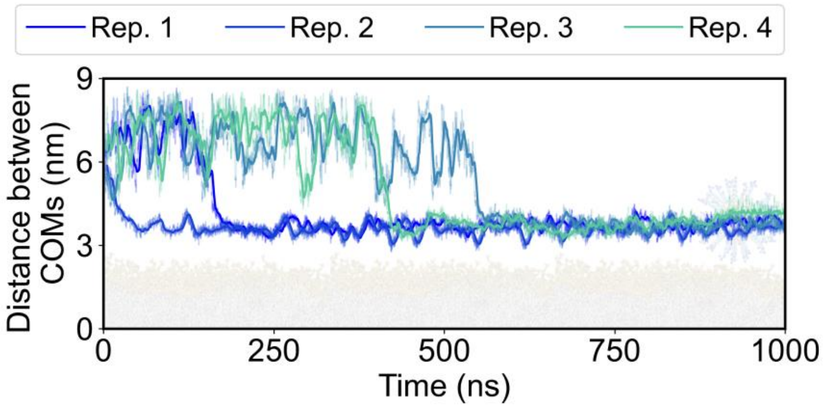

Supplementary Figure 34: Distance between the COM of the membrane and the COM of the gold atoms during the four replica simulations of **1**@AuNP interacting with a pure POPC bilayer. **1**@AuNP is the only particle to spontaneously bind to the PC bilayer even though they all explore states of sufficient proximity to the bilayer. Gold atoms are displayed in orange, sulphur in yellow, carbon in cyan, nitrogen in blue, hydrogen in white, PC headgroups in brown, and hydrophobic lipid tails in grey.

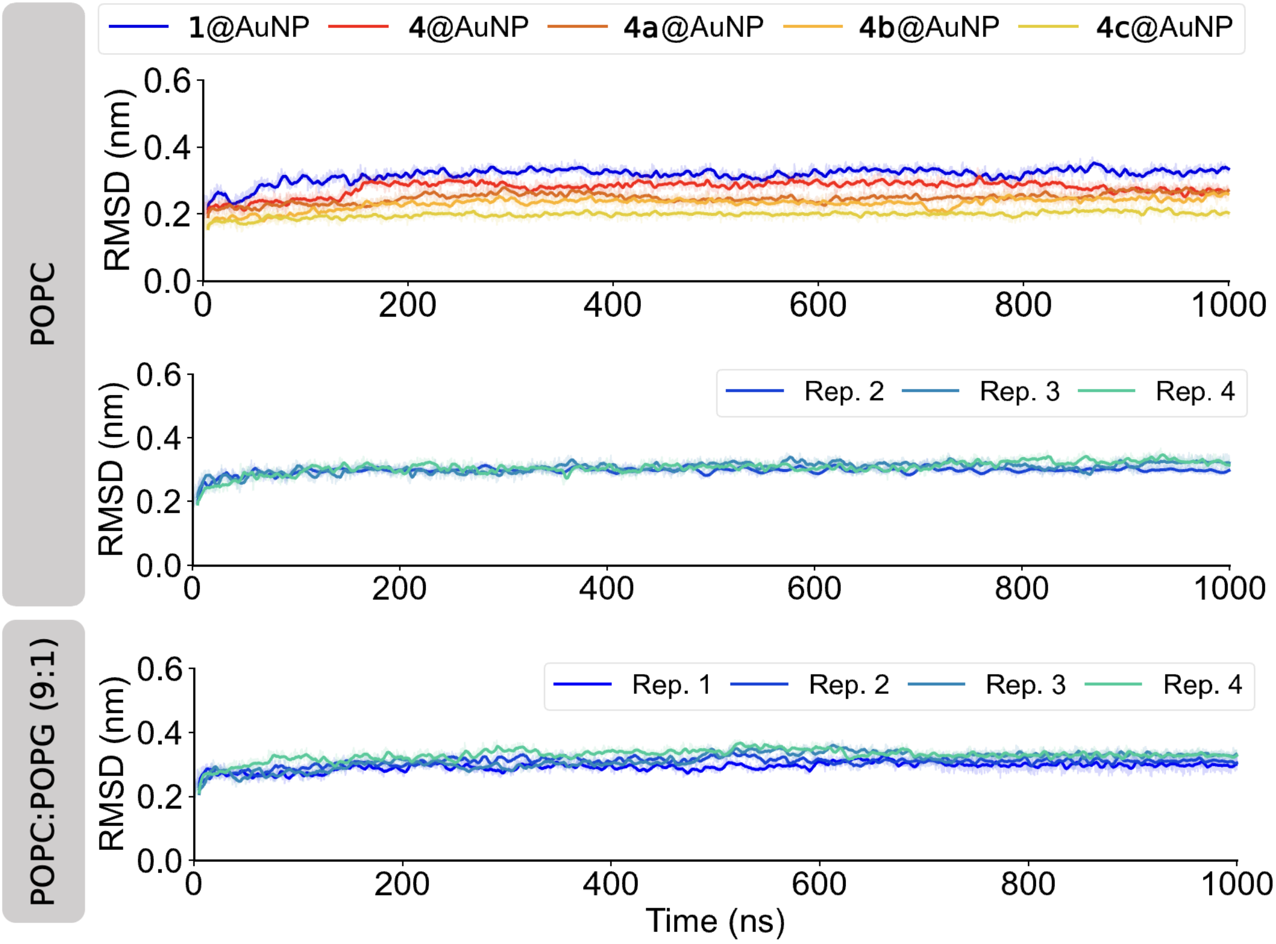

Supplementary Figure 35: Root-mean-squared deviation (RMSD) of the atomic coordinates for the AuNPs in our simulations with a membrane. The RMSD was calculated after aligning the AuNPs with their starting conformation throughout the trajectory. All the structures converge within the first 3 ns of simulation as evidenced by the flattening of the curve. The RMSD of the AuNPs was calculated for our simulations with a pure POPC bilayer (top panels) and a POPC:POPG (9:1) bilayer (bottom panel).

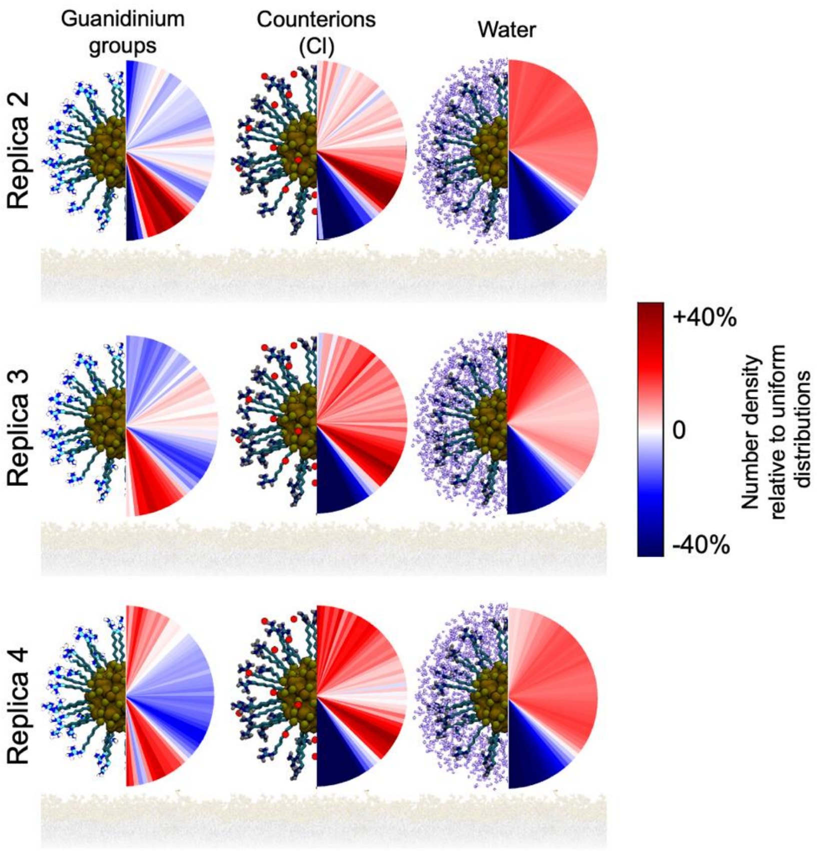

Supplementary Figure 36: Angular (polar) number density relative to a perfectly homogeneous distribution for three replica simulations of **1**@AuNP interacting with a pure POPC bilayer. In the colour bar, white indicates a perfectly uniform distribution in the spherical slice, red are highly populated regions, and blue are the least dense regions. Headgroups are shown in cyan carbons, chloride ions in red, and water molecules in purple.

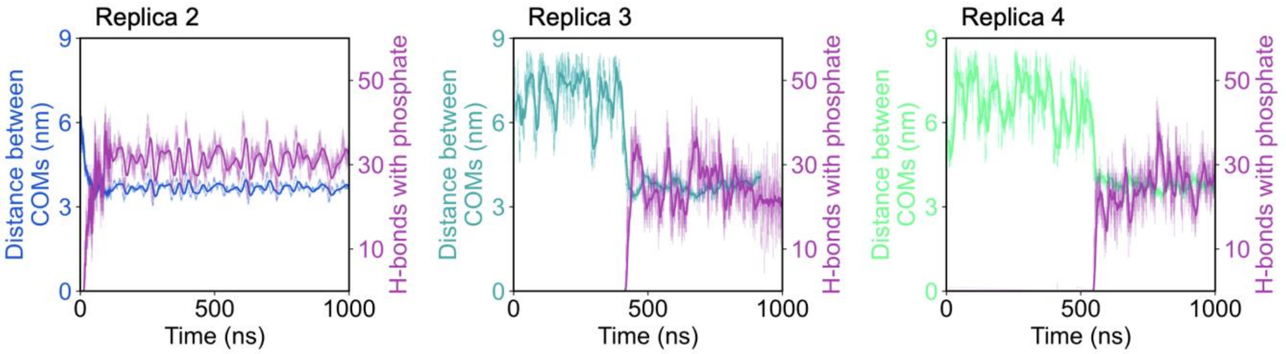

Supplementary Figure 37: Distance between the COM of the POPC bilayer and the COM of the gold atoms (blue, aquamarine, and green), as well as the number of H-bonds between **1**@AuNP and the bilayer (violet). The approach of **1**@AuNP triggers an H-bond network that stabilizes the bound complex. The results obtained for the first replica simulation can be found in Figure 9B of the main text.

		Replica 1		Replica 2		Replica 3		Replica 4	
		Water	PO_4^-	Water	PO_4^-	Water	PO_4^-	Water	PO_4^-
Before binding	ε	40	0	40	0	40	0	40	0
	$\eta^1 + \eta^2$	177	0	177	0	177	0	177	0
After binding	ε	38	4	38	3	39	3	38	3
	$\eta^1 + \eta^2$	159	23	158	26	160	22	161	21

Supplementary Table 4: Number of hydrogen bonds (H-bonds) formed between **1**@AuNP and its surroundings during the four replica simulations of **1**@AuNP interacting with a pure POPC bilayer. The table shows the number of H-bonds between i) the 60 ε nitrogen atoms in **1**@AuNP and the oxygen atoms of the solvent, ii) the 60 ε nitrogen atoms in **1**@AuNP and the phosphate group in the lipids, iii) the 120 η nitrogen atoms in **1**@AuNP and the oxygen atom of the solvent, and iv) the 120 η nitrogen atoms in **1**@AuNP and the phosphate group in the lipids.

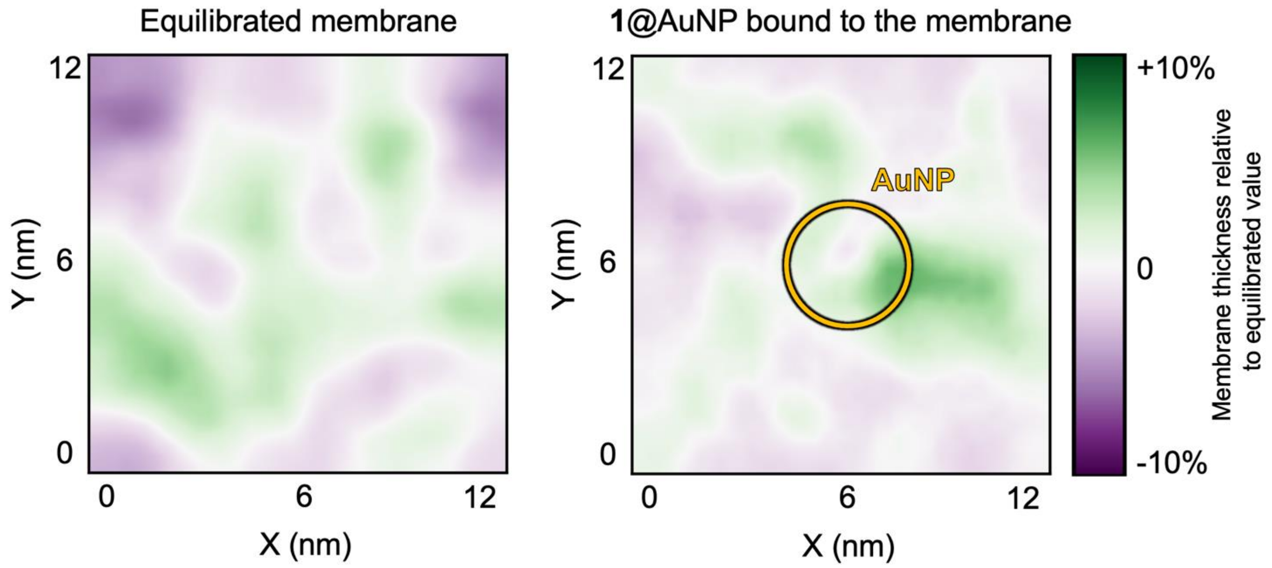

Supplementary Figure 38: Map of the membrane thickness form a top view. The unperturbed, equilibrated membrane (left panel) displayed an equilibrium value of 3.79 ± 0.27 nm. Upon the binding of **1**@AuNP in the first replica simulation (right panel), the lipids closer than 3.5 nm from the AuNP's COM on the XY plane showed a thickness of 3.83 ± 0.25 nm.

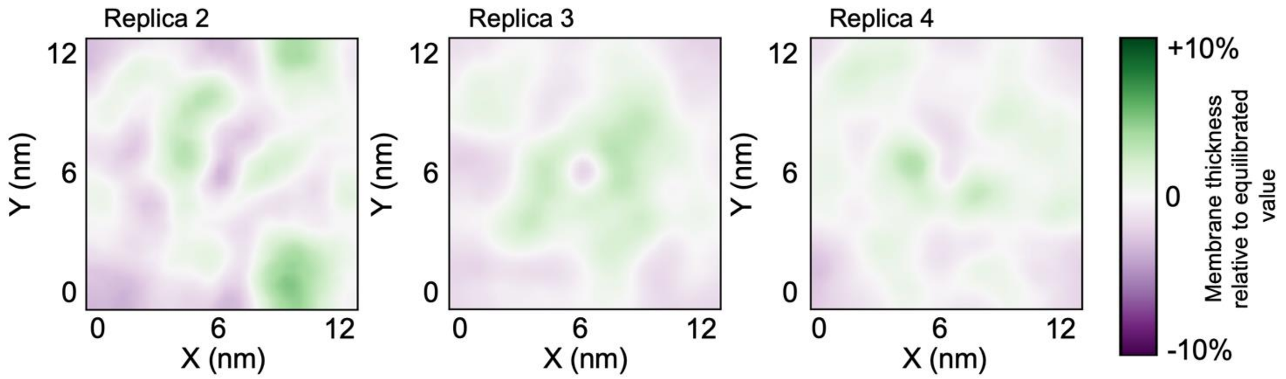

Supplementary Figure 39: Map of the pure POPC membrane's thickness from a top view. The unperturbed, equilibrated membrane displayed an equilibrium value of 3.79 ± 0.27 nm. Upon the binding of **1**@AuNP in three replica simulations, the lipids closer than 3.5 nm from the AuNP's COM on the XY plane showed a thickness of 3.79 ± 0.27 nm, 3.81 ± 0.27 nm, and 3.85 ± 0.27 nm, for replicas 2, 3, and 4, respectively.

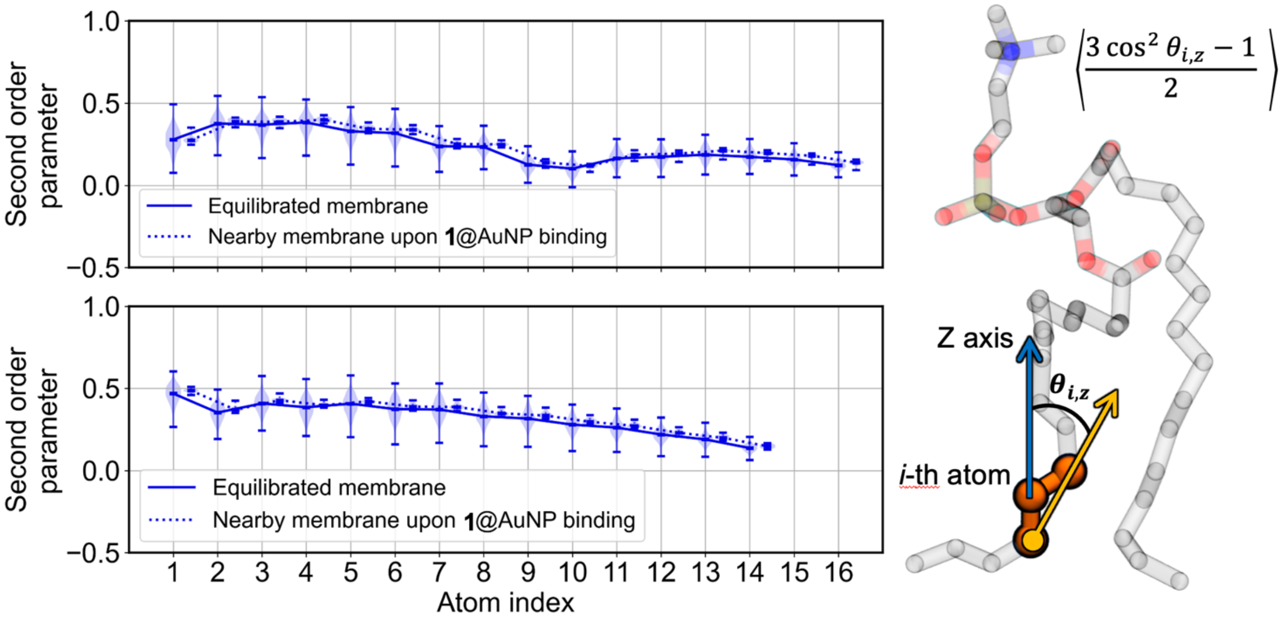

Supplementary Figure 40: Lipid order parameter for the oleoyl (OL, top panel) and palmitoyl (PA, bottom panel) tails of the POPC lipids. The order parameters of the membrane-nanoparticle complex are calculated for lipids at the contacting region as defined in the caption of Figure S29. The plots show the lipid order parameters of the equilibrated membrane (dashed lines) and the bound complex (solid lines) for the first replica simulation of **1**@AuNP interacting with a pure POPC bilayer.

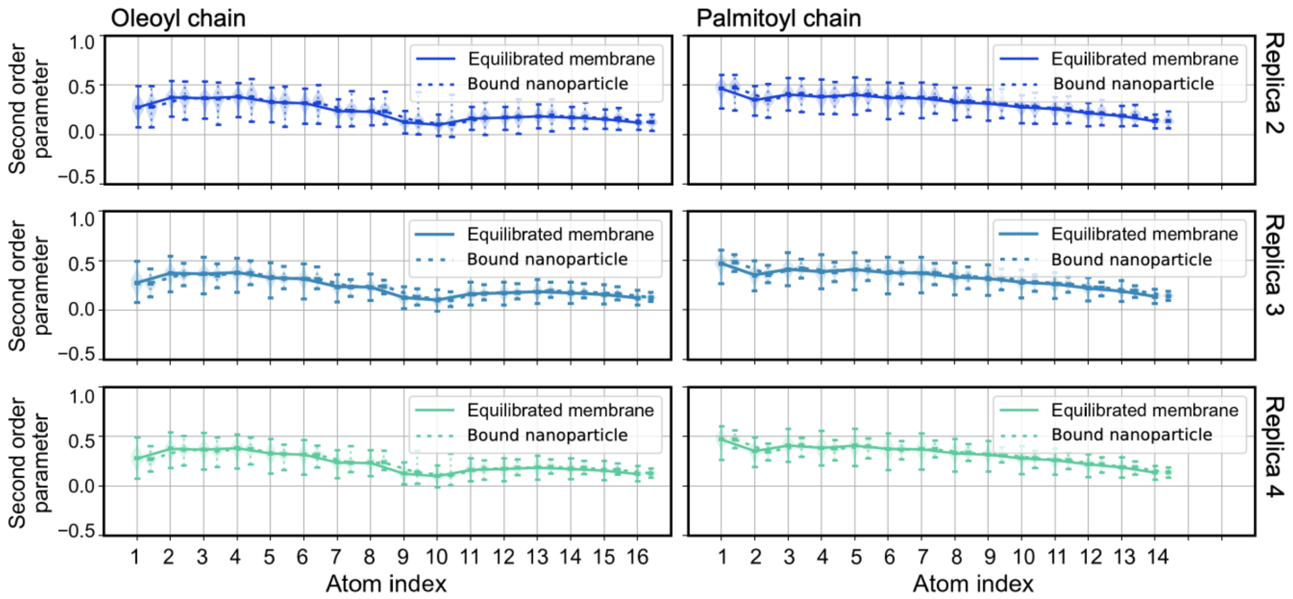

Supplementary Figure 41: Lipid order parameter for the oleoyl (OL, top panel) and palmitoyl (PA, bottom panel) tails of the POPC lipids. The order parameters of the membrane-nanoparticle complex are calculated for lipids at the contacting region as defined in the caption of Figure S29. The plots show the lipid order parameters of the equilibrated membrane (dashed lines) and the bound complexes (solid lines) for three replica simulations of **1**@AuNP interacting with a pure POPC bilayer.

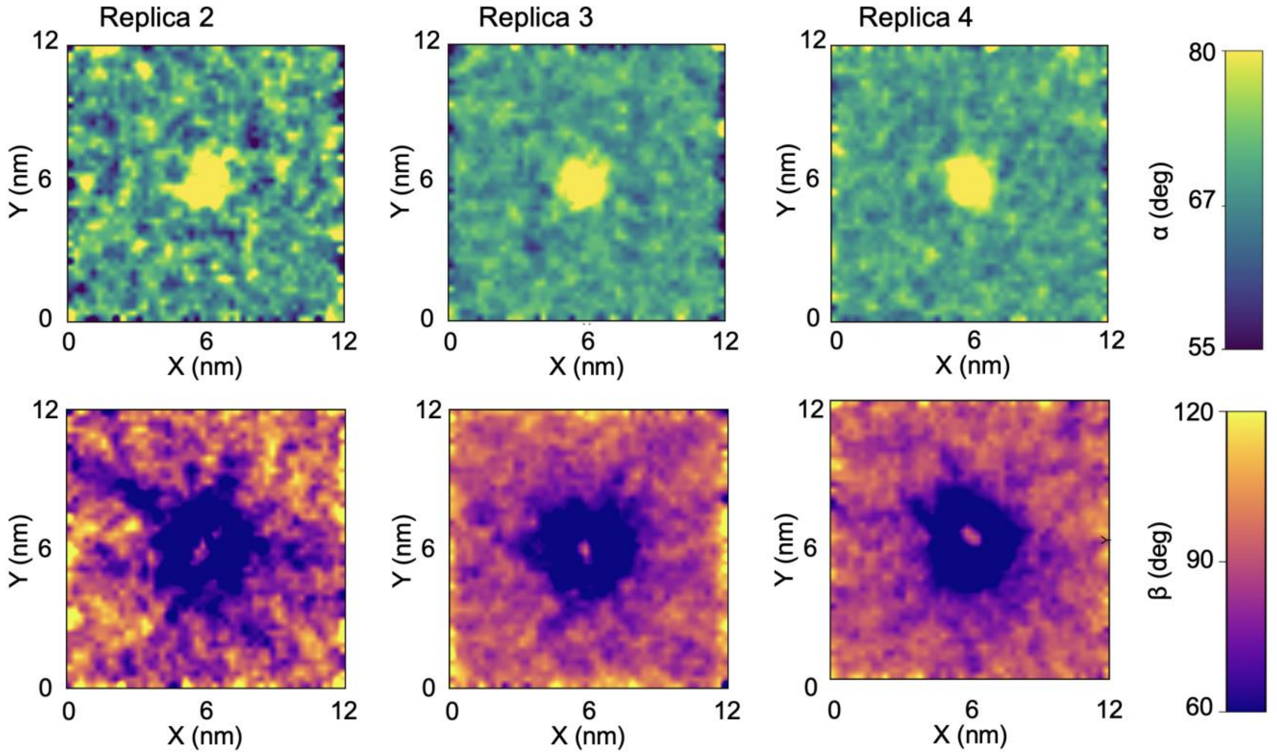

Supplementary Figure 42: Membrane deformations upon binding of **1**@AuNP to a pure POPC bilayer in three replica simulations. Map of α upon nanoparticle binding (top panels), as seen from the top (X and Y are the dimensions of the simulation box). There is a local decrease of α in the contact region. Map of β upon nanoparticle binding (bottom panels), as seen from the top. There is a local decrease of β around the contact region.

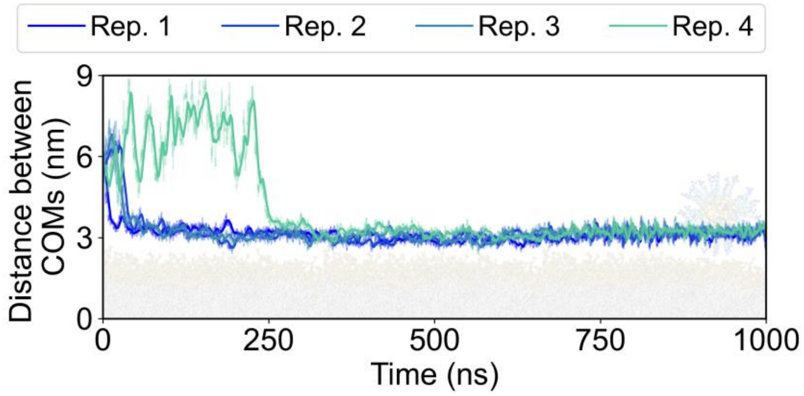

Supplementary Figure 43: Distance between the COM of the POPC:POPG bilayer and the COM of the gold atoms for the four replica simulations of **1**@AuNP interacting with a POPC:POPG (9:1) bilayer.

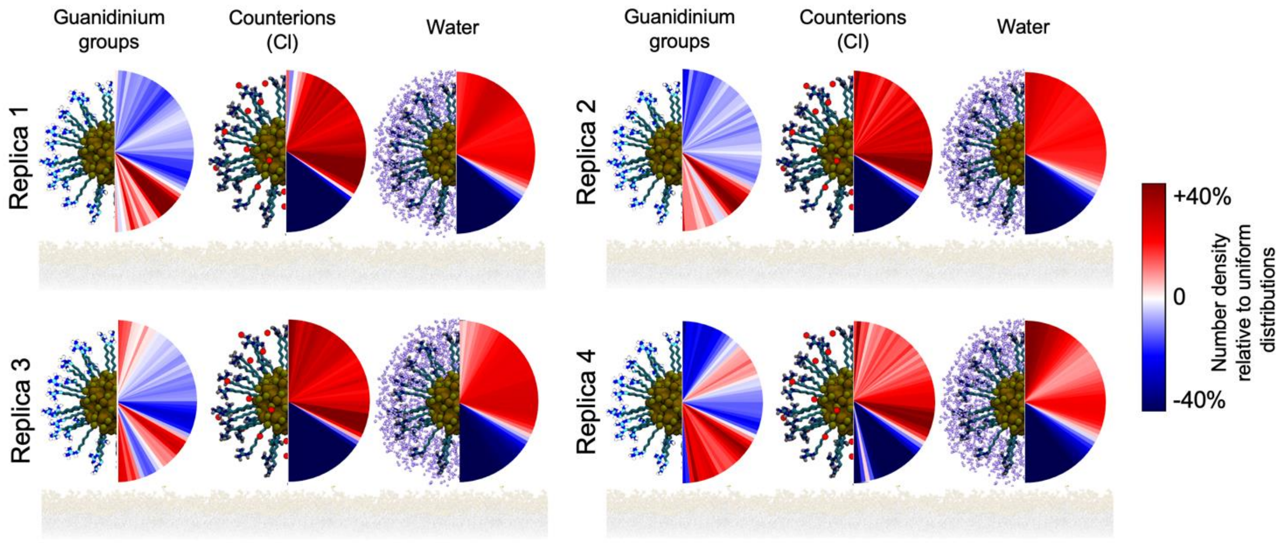

Supplementary Figure 44: Angular (polar) number density relative to a perfectly homogeneous distribution for all four replica simulations of **1**@AuNP interacting with a POPC:POPG (9:1) bilayer. In the colour bar, white indicates a perfectly uniform distribution in the spherical slice, red are highly populated regions, and blue are the least dense regions. Headgroups are shown in cyan carbons, chloride ions in red, and water molecules in purple.

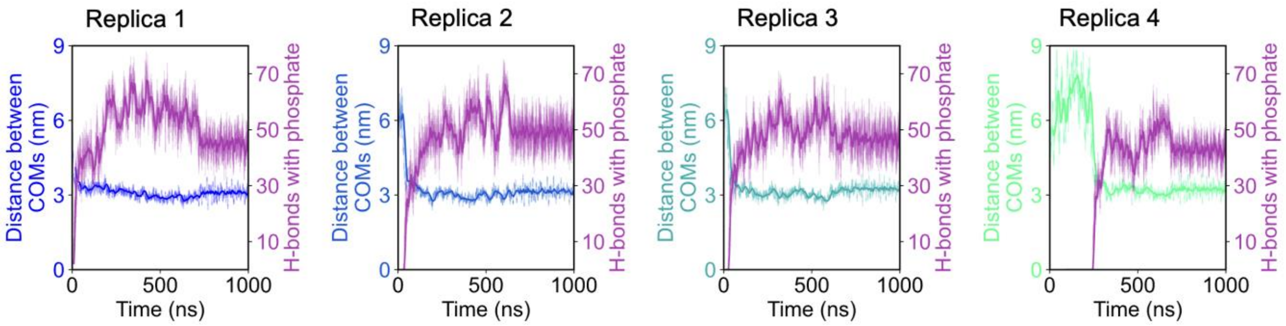

Supplementary Figure 45: Distance between the COM of the POPC:POPG (9:1) bilayer and the COM of the gold atoms (blues, aquamarine, and green), as well as the number of H-bonds between **1**@AuNP and the bilayer (violet). The approach of **1**@AuNP triggers an H-bond network that stabilizes the bound complex.

		Replica 1		Replica 2		Replica 3		Replica 4	
		Water	PO_4^-	Water	PO_4^-	Water	PO_4^-	Water	PO_4^-
Before binding	ε	40	0	40	0	40	0	40	0
	$\eta^1 + \eta^2$	177	0	177	0	177	0	177	0
After binding	ε	37	8	37	7	37	7	37	5
	$\eta^1 + \eta^2$	144	44	147	42	146	42	148	39

Supplementary Table 5: Number of hydrogen bonds (H-bonds) formed between **1**@AuNP and its surroundings during the four replica simulations of **1**@AuNP interacting with a POPC:POPG (9:1) bilayer. The table shows the number of H-bonds between i) the 60 ε nitrogen atoms in **1**@AuNP and the oxygen atoms of the solvent, ii) the 60 ε nitrogen atoms in **1**@AuNP and the phosphate group in the lipids, iii) the 120 η nitrogen atoms in **1**@AuNP and the oxygen atom of the solvent, and iv) the 120 η nitrogen atoms in **1**@AuNP and the phosphate group in the lipids.

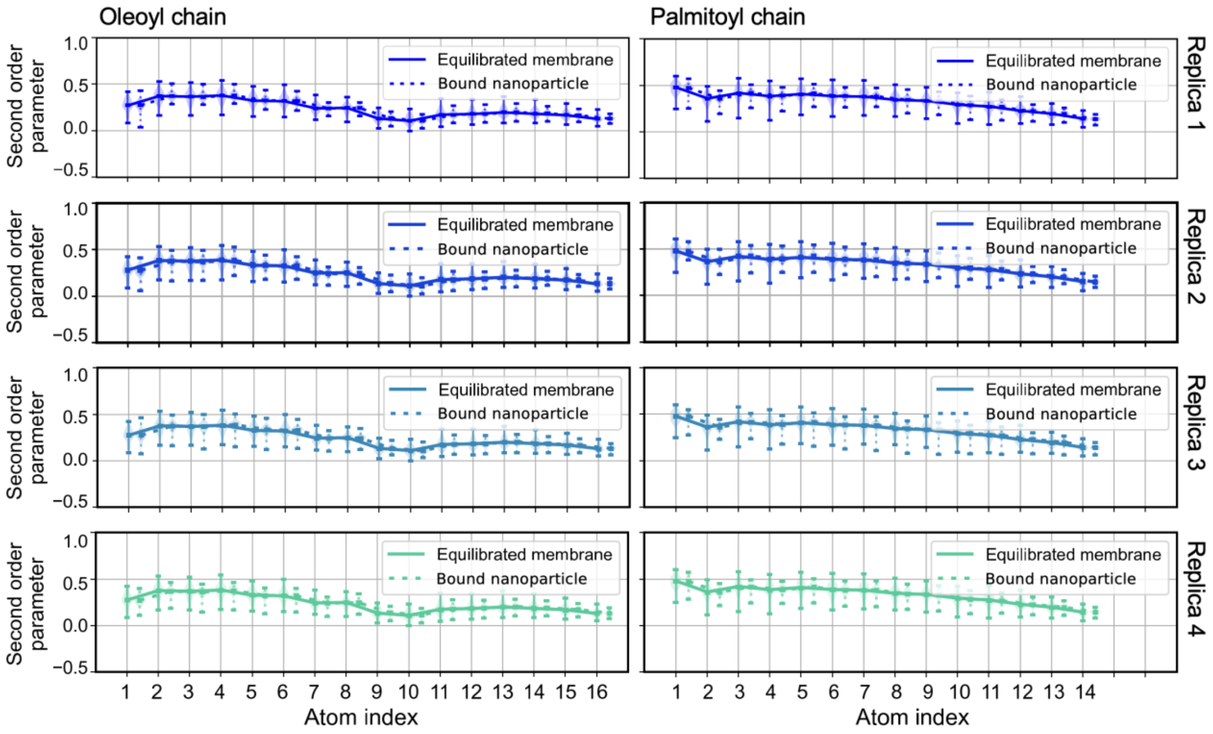

Supplementary Figure 46: Lipid order parameter for the oleoyl (OL, top panel) and palmitoyl (PA, bottom panel) tails of the POPC lipids in the POPC:POPG (9:1) bilayer. The order parameters of the membrane-nanoparticle complex are calculated for lipids at the contacting region as defined in the caption of Figure S29. The plots show the lipid order parameters of the equilibrated membrane (dashed lines) and the bound complexes (solid lines) for all four replica simulations of **1**@AuNP interacting with a POPC:POPG (9:1) bilayer.

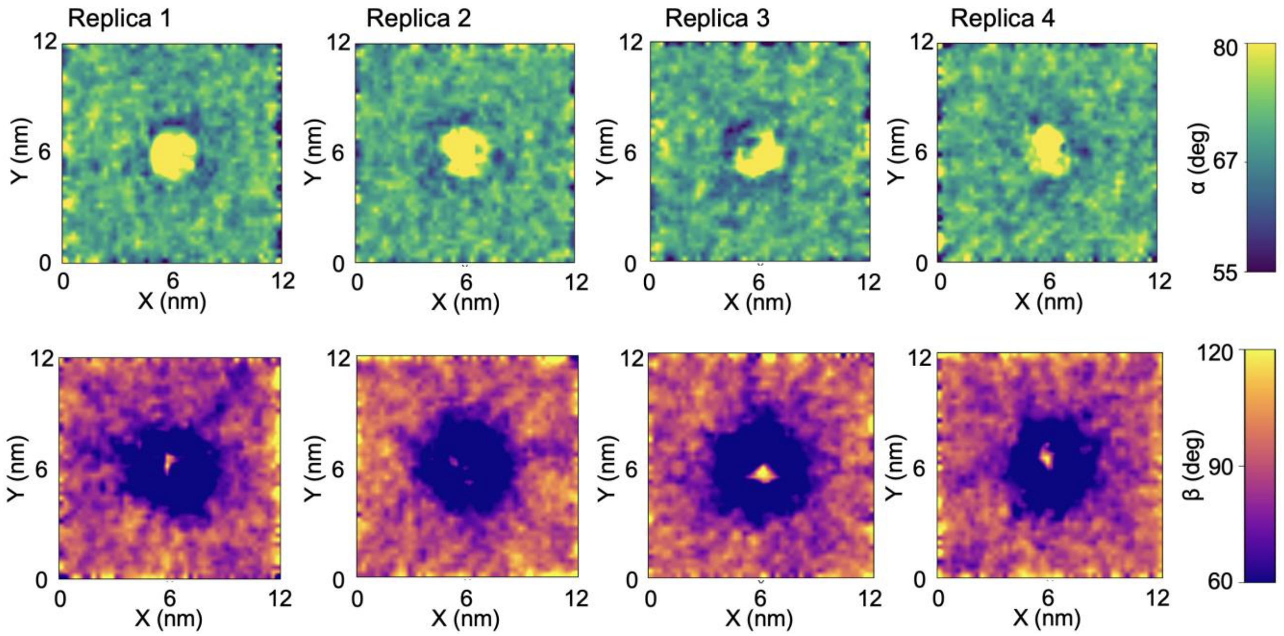

Supplementary Figure 47: Membrane deformations upon binding of **1**@AuNP to a POPC:POPG (9:1) bilayer in all four replica simulations. Map of α upon nanoparticle binding (top panels), as seen from the top (X and Y are the dimensions of the simulation box). There is a local decrease of α in the contact region. Map of β upon nanoparticle binding (bottom panels), as seen from the top. There is a local decrease of β around the contact region.

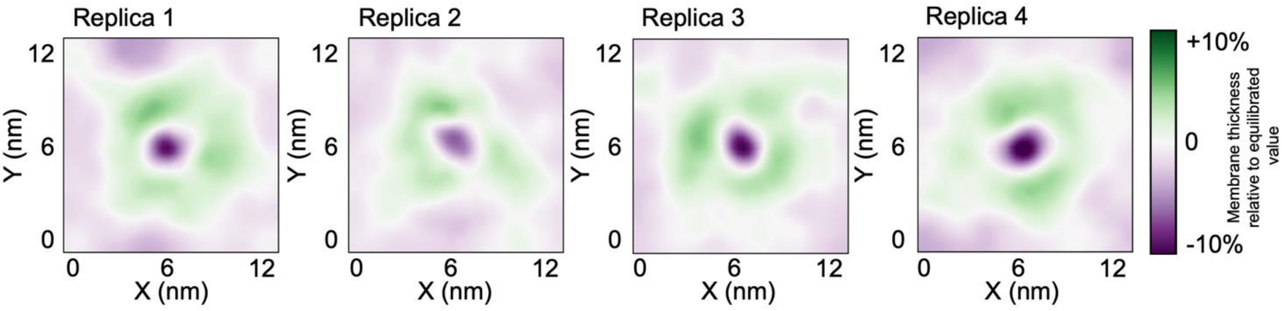

Supplementary Figure 48: Map of the POPC:POPG (9:1) membrane's thickness from a top view. The unperturbed, equilibrated membrane displayed an equilibrium value of 3.81 ± 0.26 nm. Upon the binding of **1**@AuNP in all four replica simulations, the lipids closer than 3.5 nm from the AuNP's COM on the XY plane showed a thickness of 3.84 ± 0.28 nm, 3.83 ± 0.28 nm, 3.85 ± 0.27 nm, and 3.85 ± 0.27 nm, for replicas 1, 2, 3, and 4, respectively.

5. Supplementary References

1. Bonomi, R., Cazzolaro, A., Prins, L. J. Assessment of the morphology of mixed SAMs on Au nanoparticles using a fluorescent probe. *Chem. Commun*.,2011, **47**, 445-447.
2. Sun, X., Liu, P., Mancin F. Sensor arrays made by self-organized nanoreceptors for detection and discrimination of carboxylate drugs. *Analyst*, 2018, **143**, 5754-5763.
3. Franco-Ulloa, S., Riccardi, L., Rimembrana, F., Pini, M., De Vivo, M. NanoModeler: A Webserver for Molecular Simulations and Engineering of Nanoparticles. *J. Chem. Theory Comput*., 2019, **15**, 2022-2032.
4. Heinz, H., Vaia, R. A., Farmer, B. L., Naik, R. R. Accurate Simulation of Surfaces and Interfaces of Face-Centered Cubic Metals Using 12−6 and 9−6 Lennard-Jones Potentials. *J. Phys. Chem. C*, 2008, **112**, 17281-17290.
5. Pohjolainen, E., Chen, X., Malola, S., Groenhof, G., Häkkinen, H. A Unified AMBER-Compatible Molecular Mechanics Force Field for Thiolate-Protected Gold Nanoclusters. *J. Chem. Theory Comput.*, 2016, **12**, 1342-1350.
6. Bayly, C. I., Cieplak, P., Cornell, W. D., Kollman, P. A. A well-behaved electrostatic potential based method using charge restraints for deriving atomic charges: the RESP model. *J. Phys. Chem*., 1993, **97**, 10269-10280.
7. Cornell, W. D, Cieplak, P., Bayly, C. I., Kollman, P. A. Application of RESP charges to calculate conformational energies, hydrogen bond energies, and free energies of solvation. *J. Am. Chem. Soc., 1993,* **115**, 9620-9631.
8. Vanquelef, E. *et al.* R.E.D. Server: a web service for deriving RESP and ESP charges and building force field libraries for new molecules and molecular fragments. *Nucleic Acids Res*., 2011, **39**, W511-W517.
9. Wang, J., Wolf, R. M., Caldwell, J. W., Kollman, P. A., Case, D. A. Development and testing of a general amber force field. *J. Comput. Chem*., 2004, **25**, 1157-1174.
10. Jorgensen, W. L., Chandrasekhar, J., Madura, J. D., Impey, R. W., Klein, M. L. Comparison of simple potential functions for simulating liquid water. *J. Chem. Phys*., 1983, **79**, 926-935.
11. Parrinello, M., Rahman, A. Polymorphic transitions in single crystals: A new molecular dynamics

method. *J. Appl. Phys.*, 1981, **52**, 7182-7190.

12. Jo, S., Kim, T., Iyer, V. G., Im, W. CHARMM - GUI: A web - based graphical user interface for CHARMM. *J. Comput. Chem.*, 2008, **29**, 1859-1865.
13. Skjevik, Å. A., Madej, B. D., Walker, R. C. & Teigen, K. LIPID11: A Modular Framework for Lipid Simulations Using Amber. *J. Phys. Chem. B*, 2012, **116**, 11124-11136.
14. Dickson, C. J. *et al.* Lipid14: The Amber Lipid Force Field. *J. Chem. Theory Comput.*, 2014, **10**, 865-879.
15. Hess, B., Bekker, H., Berendsen, H. J. C., Fraaije, J. G. E. M. LINCS: A linear constraint solver for molecular simulations. *J. Comput. Chem.*, 1997, **18**, 1463-1472.
16. Darden, T., York, D. & Pedersen, L. Particle mesh Ewald: An N·log(N) method for Ewald sums in large systems. *J. Chem. Phys.*, 1993, **98**, 10089-10092.
17. Hess, B., Kutzner, C., van der Spoel, D., Lindahl, E. GROMACS 4: Algorithms for Highly Efficient, Load-Balanced, and Scalable Molecular Simulation. *J. Chem. Theory Comput.*, 2008, **4**, 435-447.
18. Van Der Spoel, D. *et al.* GROMACS: Fast, flexible, and free. *J. Comput. Chem.*, 2005, **26**, 1701-1718.
19. Michaud-Agrawal, N., Denning, E. J., Woolf, T. B. & Beckstein, T. O. MDAnalysis: A toolkit for the analysis of molecular dynamics simulations. *J. Comput. Chem.*, 2011, **32**, 2319-2327.
