# Supplementary material for: Specific and nondisruptive interaction of guanidium-functionalized gold nanoparticles with neutral phospholipid bilayers
Source: Commun Chem. 2021 Jun 18;4:93. doi: 10.1038/s42004-021-00526-x (PMC9814519; doi:10.1038/s42004-021-00526-x)
Supplement: Supplementary file 2 — Description of Additional Supplementary Files [file 42004_2021_526_MOESM2_ESM.pdf]

## Description of Additional Supplementary Files

**File Name:** Supplementary Video 1

**Description**: Cryoelectron tomography video of the PC liposomes with 1@AuNPs shown in figure 6. Scale bar is 100 nm.

**File Name:** Supplementary Video 2

**Description**: Equilibrium MD simulation of 1@AuNP interacting with a pure POPC bilayer. In the 1 μs-long simulation, the nanoparticle diffuses freely in the solvent until it encounters the membrane at 165 ns. At this point, 1@AuNP binds to the bilayer irreversibly for the rest of the simulation. Similar results are obtained for our 3 replicas presenting binding times of 25, 420, and 555 ns, as reported in the main manuscript.
